# Low-Dose, Long-Wave UV Light Does Not Affect Gene Expression of Human Mesenchymal Stem Cells

**DOI:** 10.1371/journal.pone.0139307

**Published:** 2015-09-29

**Authors:** Darice Y. Wong, Thanmayi Ranganath, Andrea M. Kasko

**Affiliations:** Department of Bioengineering, Henry Samueli School of Engineering, University of California Los Angeles, Los Angeles, California, United States of America; Michigan State University, UNITED STATES

## Abstract

Light is a non-invasive tool that is widely used in a range of biomedical applications. Techniques such as photopolymerization, photodegradation, and photouncaging can be used to alter the chemical and physical properties of biomaterials in the presence of live cells. Long-wave UV light (315 nm–400 nm) is an easily accessible and commonly used energy source for triggering biomaterial changes. Although exposure to low doses of long-wave UV light is generally accepted as biocompatible, most studies employing this wavelength only establish cell viability, ignoring other possible (non-toxic) effects. Since light exposure of wavelengths longer than 315 nm may potentially induce changes in cell behavior, we examined changes in gene expression of human mesenchymal stem cells exposed to light under both 2D and 3D culture conditions, including two different hydrogel fabrication techniques, decoupling UV exposure and radical generation. While exposure to long-wave UV light did not induce significant changes in gene expression regardless of culture conditions, significant changes were observed due to scaffold fabrication chemistry and between cells plated in 2D versus encapsulated in 3D scaffolds. In order to facilitate others in searching for more specific changes between the many conditions, the full data set is available on Gene Expression Omnibus for querying.

## Introduction

Light is a stimulus now widely used in the presence of cells for biotechnology applications employing photo-chemistries. A filtered light source emits a controlled wavelength and intensity of light to trigger an environmental change affecting *in vitro* cultured cells. It is generally accepted that the shorter the wavelength (higher energy), the less compatible light may be with living systems. Visible (400–700 nm) and infrared (700 nm to 1 mm) wavelengths typically do not interact with intracellular components, although prolonged exposure may generate heat depending on the light source[1]. UV light can be divided into three categories: UVA (315–400 nm), UVB (280–315 nm) and UVC (100–280 nm). UVB and UVC light are capable of inducing direct DNA damage and are often used for sterilization[2–5], while UVA may induce indirect DNA damage through the production of free radicals given the right environment and intensity[6]. Nearly all cell-based applications of photochemistry use light sources with UVA wavelengths (or longer) because they generally balance the energy requirements to achieve photoreactions with cell compatibility. However, in-depth investigations of the biological effects of light on cells is historically in the context of solar radiation and exposure of living things to sunlight. Although many reports that describe the effect of UV light on cells exist, it is difficult to compare data from these studies due to the wide variation in procedure, light sources, cell types, and other chemical species in the experiments that may be absorbing light. The effect of UVA light for the purpose of photochemistry on cells is therefore unclear.

Despite this uncertainty, many researchers (including ourselves) use UVA light in the presence of cells because it allows precise spatial and temporal control over the physical and chemical properties of materials. Techniques such as photouncaging of small molecules[7], photopatterning of surfaces and materials[8], photoencapsulation of cells[9–12], and photorelease of therapeutics or cells[13–17] allow researchers to generate complex physical and chemical cell microenvironments that cannot be accomplished with other techniques. Light is thus a tool to initiate more complex experimental perturbations.

Numerous photochemistry studies have provided control experiments for cell viability after exposure to UVA, or at most modest immunolabeling for DNA damage-related proteins. However, no study has shown definitively the absence of other unpredictable and non-toxic changes with UVA exposure, changes that may contribute to interpretations of data. Gene arrays are an ideal, though often cost-prohibitive, technique to probe these unpredictable changes.

Typical exposure conditions for cell-based photochemistry use low-intensity long wave UV light and relatively short exposure times (365 nm, I_0_ = 5–20 mW/cm^2^, t = 2–20 min; total dose 5–10 J/cm^2^). Many studies have demonstrated cytocompatibility of these techniques through common viability assays such as the tetrazoluim bromide based MTT or MTS assays, or the calcein AM/ethidium homodimer-based Live/Dead assay [9, 18–20]. In a seminal study, Bryant, Nuttleman and Anseth reported the cytocompatability of a series of photoinitiating systems for encapsulation of NIH 3T3 cells and chondrocytes[9]. They found that nearly all chondrocytes survived encapsulation in polyethylene glycol gels using low concentrations of 2-hydroxy-1-[4-(hydroxyethoxy)phenyl]-2-methyl-1-propanone (I2959) and moderate fluxes (t = 10 min, I_0_ = 8 mW/cm^2^; 4.8 J/cm^2^ total dose). Elisseeff’s group investigated the cytocompatibility of photointiating systems over a broad range of mammalian cell types and species, and found they caused minimal cell death[20]. Interestingly, cell lines with shorter doubling times were more sensitive to the photoinitiating system, exhibiting higher rates of cell death compared to less proliferative cell lines. In addition to quantifying cell viability, a few studies have further investigated DNA damage in the presence of photoinitiators. Burdick’s group reported incidentally that large fluxes of 365 nm light (t = 30 min, I_0_ = 10 mW/cm^2^; 18 J/cm^2^) increase P53 protein immunolabelling in human mesenchymal stem cells (hMSC)[21], a potential indicator of DNA damage. Very large fluxes of UVA light (50 J/cm^2^) without photoinitiating systems are known to cause severe membrane damage to human fibroblasts[22].

While such studies indicate that UVA light appears to be generally non-toxic at reasonable fluxes (but potentially damaging at larger fluxes), no other behavioral changes have been tracked. Also, because many of these early studies were done with photoencapsulation as the primary application, the effects of light alone and the reactive species generated by light (i.e. radicals) are coupled. Photoinitiating systems used with cells usually generate reactive radical species, which may induce cell damage. It is imperative to decouple these effects since techniques such as photorelease and photouncaging do not necessarily generate reactive radical species. Additionally, unless exposure conditions are carefully regulated, heat generation may confound the results. The expanding use of long-wave UV light for *in vitro* applications demands a more thorough look at the biological effects of this type of light exposure. To address all of these issues, we characterized the effect of long wave UV-light exposure on the gene expression of human mesenchymal stem cells in the absence and presence of radicals, in both 2D (monolayer) and 3D (encapsulated) culture systems. We extended the exposure time with low intensity to represent the upper limit of fluxes used in photochemistries while minimizing heat build-up. This work decouples the effects of long wavelength UV light exposure from radical polymerization and establishes a common baseline of effects from UVA light for a more broad range of applications than just photoencapsulation.

**Fig 1:**
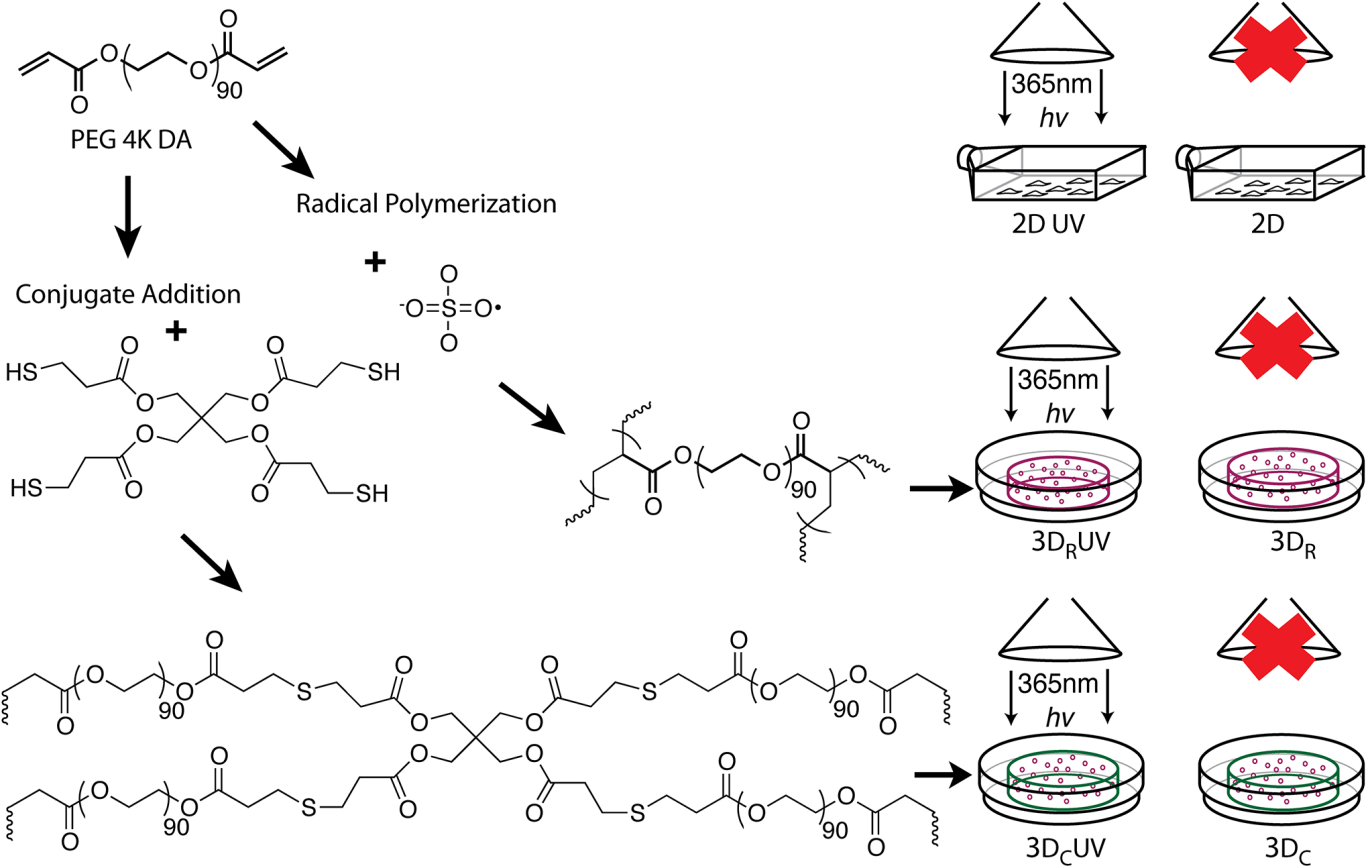
Experimental groups illustrated. A single vial of P0 hMSCs were expanded at low density to greater than 25 million cells at P1. These cells were trypsinized and a portion of them replated at 500,000 cells / 75cm^2^ flask for 2D samples. Another portion of those cells was encapsulated in poly(ethylene glycol) diacrylate (MW 4000 Da). Two encapsulation methods were used, radical polymerization with ammonium persulfate (APS) and tetramethylethylenediamine (TEMED), and conjugate addition with a pentaerythritol tetrakis(3-mercaptopropionate) (PETMP) crosslinker. Several samples of each type were created and half from each type were irradiated with a Black Ray UV bench lamp, peak wavelength 365nm.

Human mesenchymal stem cells (hMSCs) were chosen for their broad applicability and stem cell properties, a cell type widely in use but poorly characterized with regard to light exposure. These cells were received as P0 from a single donor (see S1 Note and S1 Fig for detailed discussion of experimental variability and using a single donor) and were expanded in P1 for the following culture conditions during P2: 2D (monolayer in a flask), 3D_R_ (encapsulated in poly(ethylene glycol)(PEG) via Radical polymerization), and 3D_C_ (encapsulated in PEG) via Conjugate addition (no free radicals)). Each of these three conditions (2D, 3D_R_ and 3D_C_) was irradiated with long-wave UV light (365 nm, 3.5 mW/cm^2^, 25 minutes cumulative, resulting in 5.25 J/cm^2^), for a total of six experimental groups (Fig 1). Because the 3D_C_ samples were created and collected on a different date than the 3D_R_ samples, we created a duplicate 2D group as a control between the two (2D_1_ performed with 3D_C_ and 2D_2_ performed with 3D_R_). The 2D samples were also exposed to UV light. No 2D_R_ samples were generated because the radicals kill the monolayer cells in the absence of a reactive macromer. All samples and abbreviations are summarized in S1 Table. mRNA from all the samples (performed in triplicate) was collected for gene array analysis using the Affymetrix HG-U133 Plus 2.0 Array.

We found no significant effects of long-wave UV light exposure on gene expression of hMSCs in 2D or 3D, but found the method of polymerization had a very large effect on gene expression. We used Partek Genomics Suite to identify differentially expressed genes (> 2-fold change, p < 0.05, unless otherwise noted). Gene lists were exported for functional annotation clustering and pathway analysis in two different programs, the Database for Annotation, Visualization, and Integrated Discovery (DAVID v6.7) [23, 24] and Ingenuity Pathway Analysis (IPA content version 17199142). We found no upregulated genes associated with common differentiation lineages due to UV exposure, but some are upregulated due to changes in the culture environment, either due to initial polymerization method or persistent network properties after polymerization. We observed evidence of mild DNA damage associated with radical polymerization, and robust changes between radically polymerized and conjugate addition gels in signaling pathways spanning extracellular, transmembrane, cytoskeletal, and nuclear locations.

## Results and Discussion

### Levels of gene expression cluster by culture conditions and polymerization method, not by UV exposure

**Fig 2:**
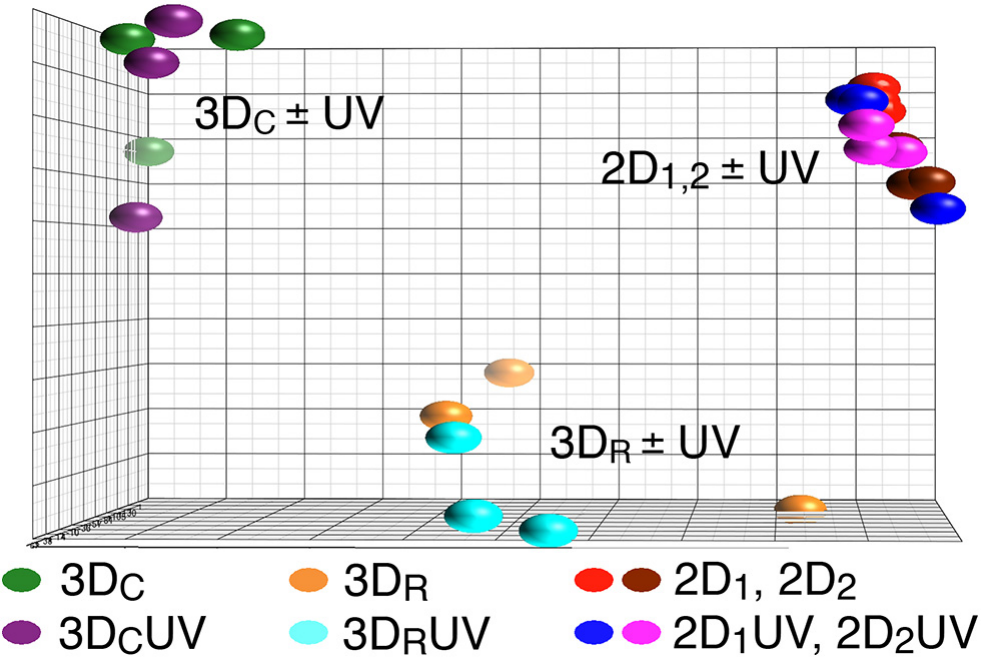
Principle components analysis shows tightly segregated clustering based on culture condition, not by UV exposure. (alternate viewing angle and axis values available in S2 Fig). PCA is a statistical analysis tool which reduces the dimensionality of data by determining the key variables resulting in differences seen between samples[25]. Each axis of this PCA map represents a linear combination of expression levels from many thousands of gene transcripts such that, combined, the maximum variation among all data points is achieved on only three axes. The result gives a visual representation of which samples behave similarly to each other by their physical closeness in three dimensions, while including information from many thousands of variables (gene expression levels). This is a bird’s eye view of the entire set of gene array data, for which absolute units and values can be considered arbitrary. The following in-depth analysis of pathway enrichment and specific gene expression provide insight into how each group differs from the others in their gene expression.

Using Principle Components Analysis (PCA), we obtain a global view of the data that suggests UV exposure does not play an important role in gene expression changes relative to other experimental conditions (Fig 2). The expression levels of each of the transcripts on the chip are collapsed into three axes to reveal similarities and differences between all the treatment samples. The cells grown in 2D are relatively uniform, regardless of expansion date or UV exposure. Each of the 3D polymerization conditions are distinctly different from each other and from 2D cells. Culture method (2D vs. 3D) and polymerization method (3D_C_ vs. 3D_R_) are the greatest determinants of gene expression, and exposure to UVA light has little to no effect on gene expression of hMSCs, regardless of culture or polymerization method. This method of viewing the data gives relative information along axes with complex meaning and somewhat arbitrary values. In the following sections we probe more deeply into specific comparisons of how each group differs from the others to rule out smaller influences from UV exposure. In particular, since the variation in the 3D samples is larger than the 2D samples, we will first look for UV specific changes across all samples, and then consider UV interactions within each culture condition.

### Specific “UV induced” gene changes are small and within error

**Fig 3:**
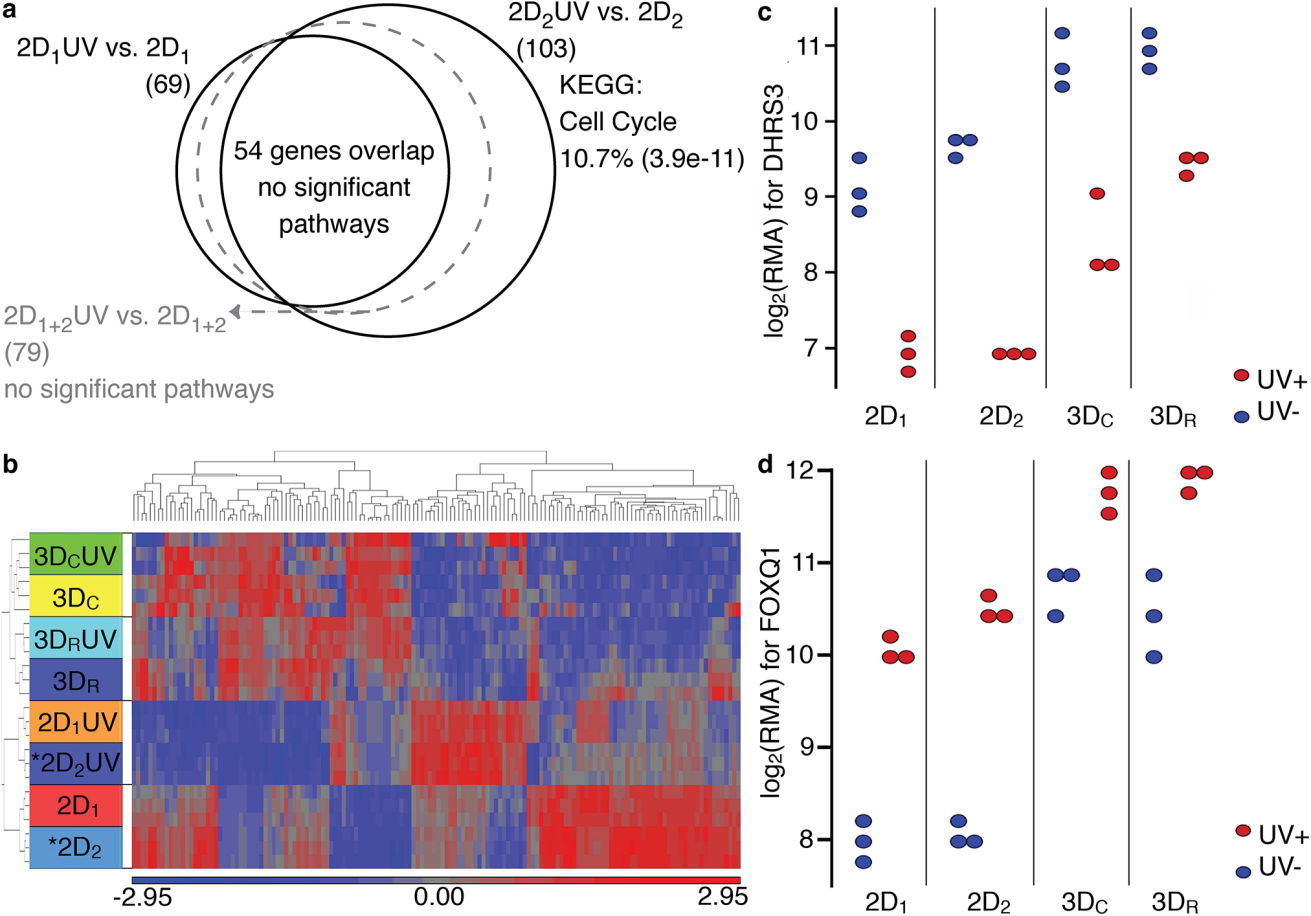
UV effects in 2D groups are insignificant. (a) This Venn diagram shows that whether combining raw data across months or using repetition power between months, no significant pathways or biological functions are found in the differentially expressed genes from the Kyoto Encyclopedia of Genes and Genomes (KEGG) or Gene Ontology (GO) except for a slight change in cell cycle. (b) The most extreme gene changes for each comparison have relatively weak fold changes, and a heat map (unsupervised hierarchical clustering) of the 148 gene transcripts (103 unique genes) differentially expressed between 2D_2_UV vs. 2D_2_ shows that none of the other UV comparisons in 3D have any differences in expression level for these genes. (red is upregulated, blue is downregulated, genes included in the heat map are those differentially expressed between denoted * groups) Also of note is the stark similarity between samples 2D_1_ and 2D_2_. The 3D groups look very similar to each other, while they are very different from the 2D groups. The most repeated changes of significance were in the genes DHRS3 and FOXQ1. Their individual scatter plots, (c) and (d), reinforce the similarities between the months as well as the direction of change. However, for so few genes and so small a change, the effects could easily be found by chance. (RMA = robust multi-array average).

While the overall picture in the PCA map indicates 365 nm UV exposure has no effect on gene expression of hMSCs at low flux, two specific genes (out of the many thousands probed) change similarly across all conditions (Fig 3, c and d). *FOXQ1* was upregulated five-fold in cells exposed to UV, and *DHRS3* was downregulated six-fold in cells exposed to UV. One must determine if these particular changes are meaningful or if they occurred by chance.

*DHRS3* helps metabolize vitamin A for different uses in the body, including but not limited to retinol in the eye[26]. It is upregulated 30–40 fold by p53 and p63 subtypes during DNA damage caused by cancer to combat the proliferation of cancerous cells[27]. *DHRS3* can be responsible for inhibiting proliferation of cancer cells, differentiation, or playing a role in apoptosis of rogue cells (see [26] for review). Aside from cancer, it also can play a role in stem cell maintenance, as proliferation and differentiation are tightly regulated in these cell types as well. *DHRS3* is mildly suppressed by UV exposure in all of our conditions, but with very low fold change relative to disease states. This could indicate an increase in proliferation or decrease in differentiation. Particularly of note, if UV light is suspected to cause isomerization of retinoic acid or DNA damage, one might expect upregulation of *DHRS3*, but here the change in *DHRS3* is opposite, decreasing slightly. That is, long-wave UV exposure does not induce the direction of change in *DHRS3* one would expect if the light was causing any damage.

*FOXQ1* is upregulated in all the UV comparisons, but never higher than five-fold. In a recent cancer study[28], comparing expression levels of the 49 forkhead transcription factors in a highly invasive breast cancer cell line (MDA-MB-231) and a noninvasive epithelial breast cancer cell line (MCF7), *FOXQ1* was 300 fold higher in the highly invasive cell line. *FOXQ1* induces epithelial-mesenchymal transition (EMT) in breast cancer cells by inhibiting E-cadherin transcription. Another report demonstrated that *FOXQ1* is responsible for regulating the transcription of *NRXN3* by binding to its promoter region, and results in increased proliferation and migration of glioma cells.[29] These two studies investigated the role of *FOXQ1* in cancer, but two reviews[30, 31] describe roles for this family in immunology and development as well. Relative to others in this family, very little is known about *FOXQ1*. In our data, *FOXQ1* is slightly increased by UV irradiation, removed from the context of cancer, this could represent a mild increase in proliferation and decrease in differentiation linked to increased stem-ness, and mirroring the effect of the change in *DHRS3*.

These two genes and their direction and magnitude of fold change indicate that there could be a mild tendency toward proliferation and migration, broad functions relevant to cell cycle and stem-ness. In this case the two differentially expressed genes cannot statistically represent any particular pathway and there is no definable effect of the UV exposure conditions on hMSCs. Considering the relative number of genes being probed, it is likely that these two genes changed in concert by chance.

### No interaction between 2D culture and UV exposure

In the 2D samples, so few genes changed after UV exposure that we could not identify any affected biological processes. There were only 54 unique overlapping genes identified in the repeated 2D_1_UV and 2D_2_UV experiments (chip contains redundant probes). For such a small number of genes, no pathways from the Kyoto Encyclopedia of Genes and Genomes (KEGG) or Gene Ontology (GO) biological processes of significance are identified in DAVID, and the numbers are similar to variations in our control comparisons (see S1 Note and S1 Fig for detailed discussion of control comparisons). Combining the raw data from all samples across months (2D_1+2_UV vs. 2D_1+2_), rather than finding their intersection, 79 unique genes are differentially expressed due to UV (Fig 3A). However, there are still no enriched pathways. Using the 103 genes from the 2D_2_UV vs. 2D_2_ comparison, we were finally able to identify a significantly enriched KEGG pathway: Cell cycle, with 10.7% of that pathway’s genes found in our gene list (p = 3.9e-11). This is still a very small number of differentially expressed genes. A heat map of this gene list from 2D_2_UV vs. 2D_2_ reveals that 2D samples between repetitions are quite similar (Fig 3B). These changes are a very small set of cell cycle genes, and likely represent normal biological variance. These gene changes are not mirrored in the 3D UV vs. no UV samples (Fig 3B).

### No interaction of 3D culture (non-radical) and UV exposure

It is widely accepted that cells behave very differently in 3D culture than in 2D culture [32–35], and investigating the effect of UV in cells cultured in 3D is especially pertinent since UV light is commonly used to generate 3D culture constructs. We used two simple 3D encapsulating conditions to allow the most basic comparisons between samples without other confounding factors. Cells were encapsulated in PEG hydrogels, without any adhesion peptides, by two different polymerization techniques, one using conjugate addition (3D_C_), the other using radical polymerization (3D_R_) initiated by redox-generated free radicals (APS/TEMED). Each of these conditions, 3D_C_ and 3D_R_, were also exposed to UV light (3D_C_ UV and 3D_R_UV). This allowed us to decouple the effects of free radicals and UV light, which are inextricably coupled for 3D hydrogels formed via photopolymerization.

In analyzing the conjugate addition gels, we found minimal changes in gene expression due to UV exposure and no identifiable pathways affected by UV exposure. Cells in the gels formed via conjugate addition (3D_C_UV vs. 3D_C_) differentially expressed only 66 transcripts due to UV exposure, comprising 48 unique genes, and 85% of them downregulated in UV compared to no UV. The fold changes ranged from -7 to +4. These changes (number of genes and fold changes) are similar in magnitude to the comparison in 2D. When all the genes were analyzed together or when split to separate lists of up and down regulated genes using DAVID, no pathways or biological functions were identified with significant p-values. (Most genes that share a network are either upregulated or downregulated together[36], and searching for significant pathways by grouping up and down regulated genes can sometimes yield findings otherwise diluted by combining all differentially expressed genes.) Because only a small number of genes are differentially expressed with only small fold changes and no pathways are identifiable, these small changes can be attributed to biological variance. Therefore, exposure to UV light has no significant effect on gene expression of hMSCs in 3D, consistent with the results for hMSCs cultured in 2D. Not only is there no damage detected, no specific biological pathways are being perturbed by UV exposure in 2D or 3D.

### Radical polymerization has strong, variable effect on gene expression, not an interaction with UV exposure

**Fig 4:**
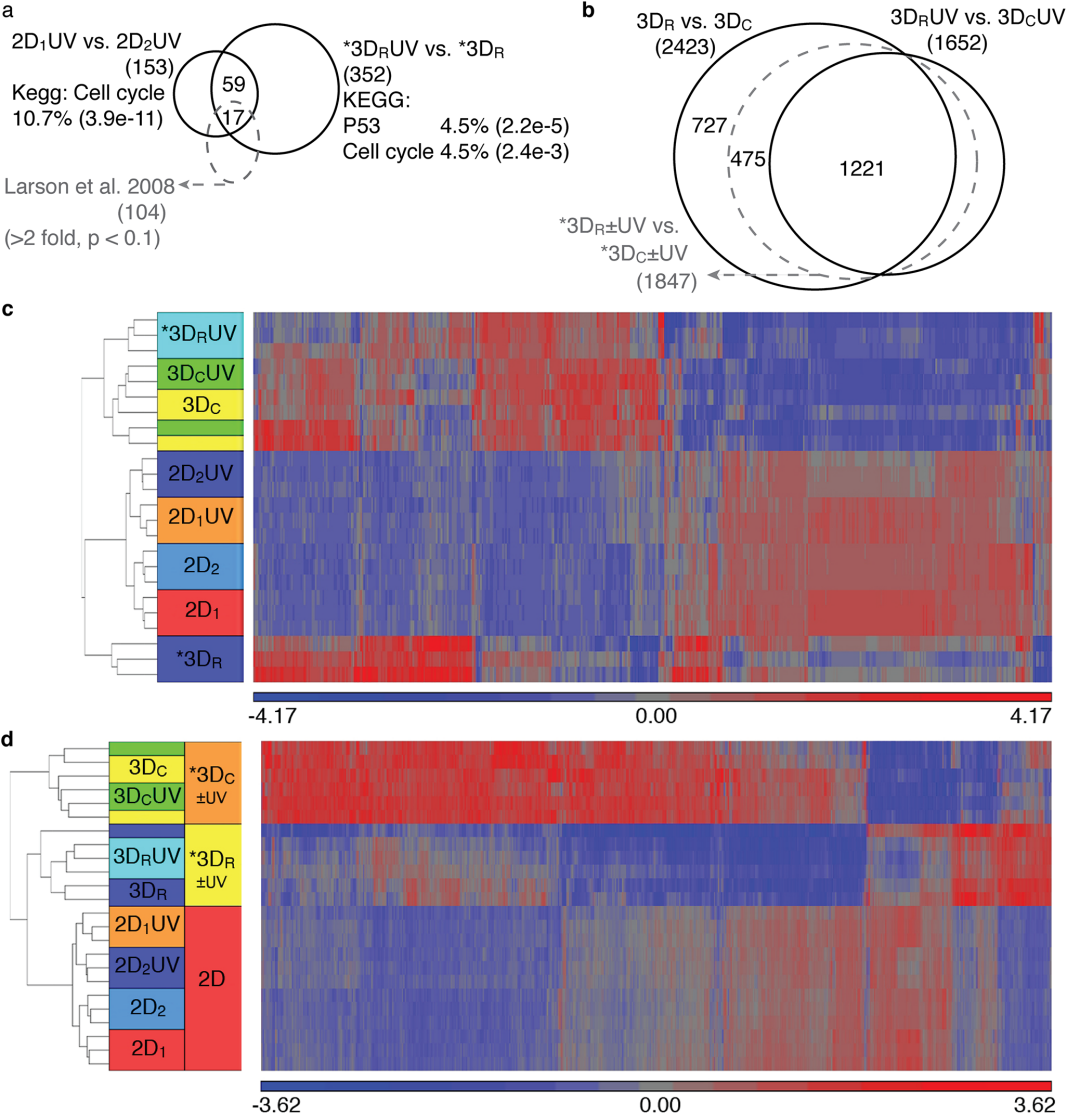
UV effects in 3D are insignificant, but environmental effects are dramatic. (a) Venn diagram showing overlapping genes between cell cycle differences and supposed UV effects in 3D. UV exposure in the radically polymerized gels results in 352 unique differentially expressed genes, which are enriched for two KEGG pathways, p53 and cell cycle, each with 4.5% of the participating genes in those pathways. These genes overlap with 50% of the 2D control genes and 17 regular cell cycle genes described by others [37]. (b) Venn diagram showing similarity of gene lists from inclusion or exclusion of UV samples when comparing 3D polymerization. Purely comparing polymerization mechanisms (3D_R_ vs. 3D_C_) yields 2423 unique genes. Making the equivalent comparison in UV (3D_R_UV vs. 3D_C_ UV) yields 1652 genes. These two comparisons share 1221 genes, all included in the 1847 genes identified by inclusion of UV samples. (c) The heat map of 507 differentially expressed genes between 3D_R_UV vs. 3D_R_ (* in (a) and (c)) (unsupervised hierarchical clustering). The 3D_R_ samples were completely separate from the other 3D groups, while all the samples for the 3D_C_ conditions were co-mingled regardless of UV condition. No differences in these genes were visible between any of the 2D conditions. (d) The heat map of 3030 differentially expressed genes between 3D_R_±UV vs. 3D_C_±UV (* in (b) and (d)) (unsupervised hierarchical clustering) reveals stark differences between polymerization conditions. 2D samples are not different for these genes. 3D samples within polymerization methods co-mingle without regard for UV exposure.

In contrast to the 2D and 3D_C_ samples, the radically polymerized 3D samples appear affected by UV exposure. These changes could represent an interaction effect between the radical polymerization method and UV exposure. However careful examination reveals that the ‘UV’ changes are more likely noise due to polymerization method and not an interaction with UV exposure. The UV comparison in radically polymerized gels (3D_R_UV vs. 3D_R_) yielded 507 differentially expressed transcripts from 352 unique genes, between hMSCs exposed to UV and those not exposed. The unsupervised hierarchical clustering (Fig 4C) for these 507 transcripts shows no correlation with other UV comparisons. With modest fold changes ranging from -9 to +6, these changes are mostly downregulated with UV exposure, and enriched most prominently for mitosis (enrichment 14.2, Benjamini 4.6e-17 using all 352 genes, similar results searching up- and down-regulated genes separately). The two KEGG pathways with p<0.01 were the cell cycle pathway and p53 signaling pathway. Each pathway was represented by 11 genes from our gene list (4.5% of the total genes in each of the pathways), four of which appeared in both of the pathways (Fig 4).

To further investigate, the fold-change cut-off in the 3D_R_UV vs 3D_R_ comparison was relaxed to 1.5 fold. With this threshold change, 2006 transcripts now meet the cut-off for differential expression instead of 507, enough now to perform a network analysis using another highly regarded database, Ingenuity™. Ingenuity Pathway Analysis (IPA) results from these 2006 transcripts similarly show the top molecular and cellular function to be Cell Cycle (p-value range 7.14e-13 to 5.19e-3, with 231 of those genes falling into the cell cycle category). Additionally, IPA finds that *TP53* is the most significant upstream regulator and is activated in the UV condition compared to no UV (p = 3.72e-25). Upstream regulators are identified in IPA (but not DAVID) based on the up- or down-regulation of genes that are directly affected by a particular molecule’s actions. While *TP53* (the gene that encodes p53) was the most significant upstream regulator, the p53 signaling pathway was only insignificantly identified (p = 0.22, ratio = 0.093), no UVA- or UVC- induced MAPK signaling was identified, and UVB-induced MAPK signaling was insignificantly identified (p = 0.40, ratio = 0.086).

Though p53 is well known to regulate DNA repair in response to short-wave (254 nm) UV radiation [38](by halting the cell cycle to allow for repair) there are no other strong indicators that DNA damage is present in this experimental condition. p53 binds to DNA and is an important regulator of the cell cycle (reviewed in [39] and [40]) in all cell types, including stem cells). EMT plasticity is also regulated by p53 [41]. The modest indications of cell cycle and p53 pathways may also in part reflect differences in cell cycle during sample collection. In fact, of the 153 genes differentially expressed between presumably identical samples 2D_1_UV and 2D_2_UV, (which overwhelmingly represent differences in mitosis between the samples), 50% of those genes can be found differentially expressed in the comparison of 3D_R_UV vs. 3D_R_ (Fig 4A). 17 of those overlapping genes were also identified as fluctuating genes in the independent study of cell cycle phase (in 2D)[37]. Thus there is strong evidence that many of the gene changes are related to cell cycle phase and are not necessarily linked to DNA damage. We see evidence that these doses of light are not damaging (confirming numerous control/viability experiments from diverse photochemistry researchers[9, 19, 21, 42, 43]), but also evidence that cell cycle changes are happening. The origins and consequences of these cell cycle changes are critical to interpretation of any experiments using UVA exposure.

The small perceived effect between 3D_R_UV vs. 3D_R_ samples could theoretically be attributable to the effect of UVA radiation on the initiating species (APS/TEMED), an interaction observable only when UVA exposure is paired with radical polymerization. However, the 507 genes differentially expressed in 3D_R_UV (versus 3D_R_) show patterns of expression similar to the 3D_C_ and 3D_C_UV samples (Fig 3C). Because the 3D_R_ samples after UVA exposure become more similar to the 3D_C_ samples which have no radical species exposure, we believe the UVA light is not accelerating or amplifying any effects of the radical polymerization technique. Since the logical interaction of UVA exposure and radical initiation is not supported, we entertained the possibility that these changes are an artifact of larger variations due to radical polymerization itself.

In order to evaluate the import of UVA exposure, we analyzed differences in gene expression between polymerization methods both including and excluding the UV data. First we determined the differential expression between radical polymerization and conjugate addition (*3D_R_±UV vs. *3D_C_ ±UV) by including UV samples for each polymerization method. The 3030 differentially expressed genes (Fig 4D) show that polymerization method is very strongly influencing many more genes than the hundreds that changed based on UV exposure alone. We then compared those results to 3D_R_ vs. 3D_C_, excluding UV samples. When evaluated with UV data, the differentially expressed genes overlap considerably with the intersection of the two separated comparisons 3D_R_ vs 3D_C_ and 3D_R_UV vs. 3D_C_UV. Identities of enriched pathways in these gene lists were not considerably affected by inclusion or exclusion of UV samples (S2 Note and S2 Table). Thus UV effects in 3D are globally insignificant because inclusion or exclusion of UV samples makes very little difference when comparing radical polymerization to conjugate addition.

The global view overlooks small changes in a few genes, but specific genes are known to be affected by UVA radiation and must be examined. The most extensive research on gene expression changes has been conducted on dermal fibroblasts or keratinocytes because of interest in the skin’s response to UV radiation. Because of this interest, the typical fluxes for these studies represent exposure conditions in full sunlight for 1–2 hours, and are in the range of 10–50 J/cm^2^, much higher than necessary for *in vitro* photoreactions. UVA induced damage in dermal fibroblasts has been documented and attributed to singlet oxygen created by UV energy (30 J/cm^2^, reported to be non-toxic to dermal fibroblasts by MTT assay). Major immediate changes in p38 and JNK activity result only if the singlet oxygen is generated intracellularly, not extracellularly [6]. Many other genes upregulated in dermal fibroblasts or keratinocytes exposed to UVA radiation from 10–50 J/cm^2^ have been identified, including heme-oxygenase[44], *ICAM-1*[45], *MMP-1*[46]. Transcription factors *NF-KappaB*[22, 47], AP-1[48–50] and *AP-2*[45], and *STAT1*[51] and *STAT3*[52] are also activated in dermal fibroblasts by UVA/singlet oxygen. Of these typical UVA-associated gene changes, only *STAT3* and *MMP-1* were differentially expressed in the 3D_R_UV vs. 3D_R_ comparison for hMSC. *STAT3* had a -3.4 fold decrease due to UV (p = 7.9e-5), which is contrary to the reported effect of UVA-induced damage, while MMP1 increased 3.02 fold (p = 3.6e-3). UV exposure in radically polymerized gels did not induce measurable UVA-associated gene changes in hMSC.

Because UVA light at high fluxes may induce the formation of reactive oxygen species in situ, causing cellular damage, we compared the 3D_R_ and 3D_C_ samples, as this comparison highlights the presence of radicals during the polymerization step. *STAT3* increased 6.48 fold in the 3D_R_ samples (p = 3.5e-8), and three of the eight genes associated with *AP-1* were also upregulated with radical polymerization: *FOSB*: 3.9 fold (p = 7.2e-4), *JUN*: 2.39 fold (p = 1.5e-8), *JUNB*: 2.2 fold (p = 2.5e-12). This indicates that exposure of hMSC (cells not naturally exposed to light) to polymerizing radicals results in modest, yet real, changes in gene expression categorized as UVA-related damage and known to result from singlet oxygen. These changes were not observed in hMSC with exposure to UVA light alone.

Taken together, these comparisons imply that the doses of UVA light used in our experiments are not sufficient to generate reactive oxygen species, reinforcing the notion that moderate doses of UVA light are innocuous to cells. Intriguingly, while researchers are not typically as concerned about the effect of the initiating radicals on cells, it is clear that hMSCs are affected by these extracellular reactive species.

### UVA exposure does not induce differentiation

Photoencapsulation, photodegradation and photouncaging/release are widely used techniques for creating well-controlled synthetic microenvironments to study cell differentiation. In order to use these synthetic microenvironments to study and guide hMSC differentiation, they should not inherently induce differentiation down a specific pathway in the absence of applied signals–that is, these hydrogels should represent a “blank slate” to the cells, or at least their baseline influence should be known. It is therefore important to determine if either light or synthetic microenvironment are potential variables in influencing cell differentiation. We have shown in this report that exposure to small doses of UVA light has no discernible impact on gene expression of hMSCs either in 2D or encapsulated in 3D hydrogels. To more closely examine whether or not the hydrogels themselves induce differentiation, we compared our results to previously published gene array data for hMSCs cultured with differentiation media in 2D [53]. That review summarized the major genes upregulated for different lineages: osteogenic, chondrogenic, adipogenic, and neurogenic. Cross-referencing Entrez Gene numbers with several of our own comparisons, we find very few overlapping genes, and some in the opposite direction (S5 Table). The review listed nine osteogenic, five chondrogenic, ten adipogenic, and six neurogenic genes which were upregulated in hMSC after treatment with differentiation medium. We found two of nine osteogenic genes, one of five chondrogenic genes, two of ten adipogenic genes, and two of six neurogenic genes either up- or down-regulated with weak fold changes and mildly significant p-values. The differences in gene expression were mostly observed between the different polymerization methods (3D_C_ vs. 3D_R_) and any changes due to UV exposure were either downregulated or insignificant.

### Real differences result from polymerization method, reflecting radical exposure and network differences

Because of the minimal influence from UV exposure, we used the power of increased replicates by including UV samples but treating them only by polymerization method. Relevant networks, functions and upstream regulators identified in IPA for the 3D_R_±UV vs. 3D_C_±UV comparison using 3030 genes (> 2-fold, p<0.05) are summarized in Table 1. Most of the gene changes can be associated with cell development, growth and proliferation, but two other processes that seem to be present are mild DNA damage and considerable changes in cell-environment interactions with the microenvironment.

**Table 1:** IPA summary for 3D_R_±UV vs. 3D_C_±UV, (>2fold, p<0.05).

Level of evaluation	Specific categories	Statistical measure^a^
**Top Networks**	RNA Post-transcriptional Modification, DNA Replication, Recombination and Repair, Antimicrobial Response	44^b^
	Cellular Assembly and Organization, Cellular Function and Maintenance, Molecular Transport	37^b^
**Molecular and cellular functions**	Cellular Development	3.1e-14–8.6e-4 (537)^c^
	Cellular Growth and Proliferation	3.1e-14–8.6e-4 (560)^c^
	Cell Cycle	1.0e-12–9.6e-4 (280)^c^
	Cellular Assembly and Organization	1.6e-12–9.5e-4 (338)^c^
	Cellular Function and Maintenance	1.63–12–9.4e-4 (334)^c^
**Upstream Regulators**	TP53 (tumor protein p53)	5.0e-25 (-0.727, not predicted)^d^
	TGFB1 (transforming growth factor beta-1)	2.9e-18 (3.344, activated)^d^
	Beta-estradiol	3.6e-18 (2.121, activated)^d^
	PDGF BB (platelet derived growth factor-BB)	7.5e-16 (2,164, activated)^d^

^a^IPA analysis returned several categories of known networks, molecular and cellular functions, and upstream regulators that could be considered important differences between radical polymerization and conjugate addition. This table summarizes the specific changes between these two polymerization groups at each level of evaluation (whole network activity, group function, specific protein activation) and gives the relative strengths of these components in the relevant statistical measure.

^b^IPA network score: For networks, the IPA network score is a relative measure of relevance, with the two highest scores reported.

^c^ p-value range (# molecules): For molecular and cellular functions, each function, such as “Cellular Development”, represents a combination of lower level functions, each with a p-value. Thus the significance of these higher level functions is given as a range that covers the p-values of the lower level functions. The # molecules is the number of user input dataset molecules associated with that higher level function.

^d^ p-value (activation z-score, prediction): For individual upstream regulators, the expected cascade of transcriptional changes in downstream molecules is evaluated to determine if a particular upstream regulator is acting, and directions and magnitudes of particular changes can be used to determine the activation z-score. Positive z-scores above 2 predict activation while negative z-scores below -2 predict inhibition.

**Fig 5:**
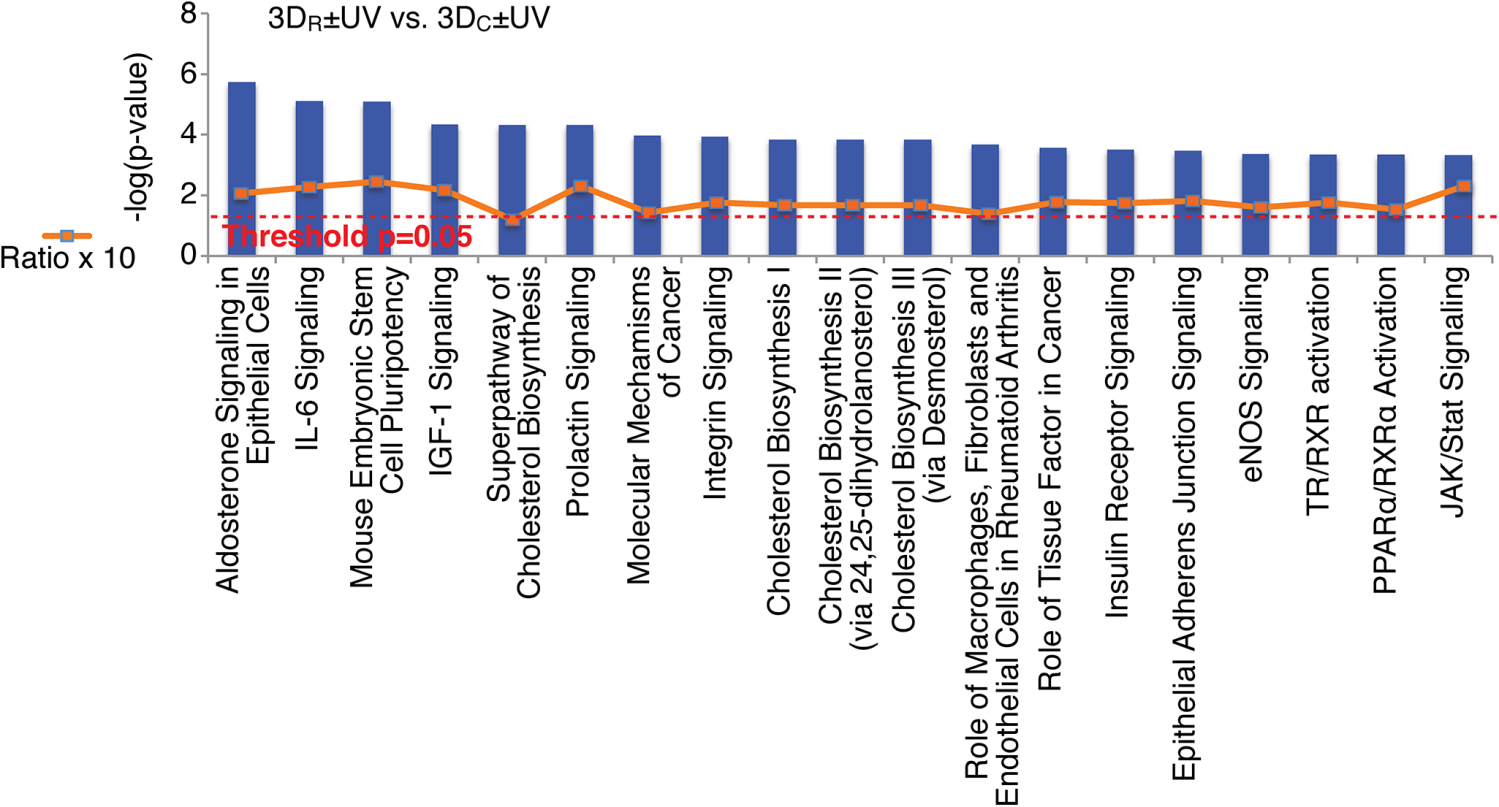
The top 20 canonical pathways identified from the 3D_R_±UV vs. 3D_C_±UV comparison ranked by p-value. Red dotted line marks p-value threshold,-log(0.05) = 1.3. The ratio of genes from our gene list found in the pathway to the total number of genes in the pathway is given as ratio x 10, and marked as the orange squares and line, and fluctuate between 15% and 25%.

Many of the most significant canonical pathways enriched between 3D_R_±UV vs. 3D_C_±UV, not surprisingly, fall within the categories of receptor signaling and the cell’s communication with and reaction to the extracellular environment (Fig 5, complete listing in S3 Table). Integrin signaling (p = 0.000115, ratio = 0.183), JAK/STAT signaling (p = 0.000468, ratio = 0.239), IL-6 signaling (p = 0.00000759, ratio = 0.234), and ERK/MAPK signaling (p = 0.00933, ratio = 0.168) are all very important in transmembrane and ECM signaling resulting in transcriptional regulation. Whereas most differences in the UV comparisons centered around cell cycle stage and mitosis, these pathways are focused on extracellular interactions. These changes indicate that cells may be able to sense small microstructural differences between the 3D_R_ and 3D_C_ gels. The two different chemistries (3D_R_ versus 3D_C_) result in hydrogel networks with some microstructural differences in the network mesh because the 3D_R_ networks likely incorporate more macromer chains per crosslink point than the 3D_C_ network, which can incorporate a maximum of four chains per crosslink site (Fig 1).

Another area dramatically affected is the cholesterol and steroid hormone related synthesis pathways, as four of the top 20 canonical pathways are related to cholesterol synthesis. Four major genes necessary for cholesterol synthesis[54] are all lower in 3D_R_±UV than 3D_C_±UV. Given here are their gene symbols, fold changes, and p-values. *HMGCS1*: -4.31(p = 4.49e-11), *HMGCR*: -2.57(p = 2.9e-10), -2.77(p = 3.13e-10), *DHCR7*:-2.41(p = 4.82e-8), -2.63(p = 3.84e-9), and *SQLE*: -2.38(p = 2.09e-8), -2.62(p = 1.00e-6).*)*. Cholesterol makes up a significant component of cell membranes and plays important roles in a number of functions related to the cell membrane and signaling (reviewed in [55–59]). Thus, increased cholesterol synthesis can be associated with stress response and membrane damage. The expression of each of the listed cholesterol genes is lower in the 3D_R_ samples (compared to 3D_C_). In addition to cholesterol synthesis (which is also related to hormone synthesis), the *RICTOR* gene is higher in 3D_C_ samples (2.33 fold, p = 1.0e-7, 2.39 fold p = 1.2e-7). *RICTOR* is a subunit of *mTORC2*, which regulates cell growth and survival in response to hormone signals, and regulates the actin cytoskeleton. Particularly in bone-derived mesenchymal stem cells, *mTORC2* and *RICTOR* play an important role in lineage specification towards osteogenic (and away from adipogenic) differentiation in response to mechanical activation[60]. These gene changes are direct evidence of the importance of extracellular environment on fundamental cell behavior and differentiation of stem cells. Differing network properties between differently fabricated hydrogels, such as density, stiffness, and adhesion molecules (or lack thereof), are the focus of many recent efforts to elucidate the mechanism of soft substrate/cell interactions for hMSC and other cell types[61–65]. A detailed analysis of the changes in gene expression between different hydrogel networks and between 2D and 3D cultures from our experiments is outside of the scope of this paper. However, this collection of gene array data can provide a highly focused starting point for researching more detailed mechanisms.

Several identified pathways in the top 100 for radical polymerization (S3 Table) are associated with DNA or cell damage such as UVA-induced MAPK signaling (p = 0.0195, ratio = 0.163), UVB-induced MAPK signaling (p = 0.0389, ratio = 0.172), UVC-induced MAPK signaling (p = 0.282, ratio = 0.136), p53 signaling (p = 0.00363, ratio = 0.176), and cell cycle G2/M DNA damage checkpoint regulation (p = 0.331, ratio 0.184). Though p53 signaling is identified as a perturbed canonical pathway, directions of fold change for its immediately affected molecules results in an activation z-score of -0.727 (Table 1), which does not allow a prediction of activation or inhibition (positive z-scores above 2 predict activation, negative z-scores below -2 predict inhibition). While some of these damage pathways are not statistically significant, most were not identified at all in the IPA analysis of the UV effects in the 3D_R_UV vs. 3D_R_ comparison, even with a less stringent fold cut-off (1.5 fold) (S4 Table).

Specific genes, *CHI3L1* and *HSPA6*, were affected significantly by polymerization method but had the opposite reaction to UV exposure (S3 Note and S3 Fig). Thus we were able to identify some pathways of DNA and cell damage due to free radical presence in the 3D_R_ samples compared to the 3D_C_ samples, while not for UV exposure. This is consistent with the previous analysis of specific genes associated with UVA exposure and dermal fibroblasts that indicates that the dose of UVA light in these experiments is not sufficient to generate cell-damaging radicals on its own.

## Conclusion

In summary, we have shown that long-wave UV (365 nm) at intensity and duration similar to commonly used conditions for biomaterials applications creates few changes in gene expression, and those changes are easily comingled with variations in cell cycle phase. At this flux, UVA exposure does not initiate common UVA-associated repair mechanisms, or gene changes associated with common *in vitro* differentiation protocols. In contrast, cells exposed to polymerization techniques using radicals (versus conjugate addition) exhibit significant differences in gene expression which include genes related to signal transduction from the extracellular environment, DNA damage from the presence of radicals, and cell cycle differences that likely stem from the previous two. The most significant differences were found as a result of culture method, in other words the persistent extracellular environment. Comparing gene changes to potential markers of differentiation, we found none would be suspected due to long-wave UV exposure, while a few could be attributed to extracellular environment, specifically osteogenic, adipogenic, and neurogenic differentiation. Our analysis concludes that differentiation or significant gene expression changes are due, not to long-wave UV exposure, but to persistent environmental conditions. Those researchers seeking to use long-wave UV light should be conscious of the fluxes used to prevent damage, but otherwise unexpected changes are most likely due to experimental conditions beyond the light exposure. This data set is relevant to many researchers interested in not only UV exposure, but also 2D/3D effects and 3D hydrogel network differences. Gene array data can be cost-prohibitive for many interested parties to perform on their own. Sharing this data on Gene Expression Omnibus should help others to focus their efforts at investigating diverse mechanisms in hMSC.

## Materials and Methods

### Chemicals

Acryloyl chloride (Alfa Aesar, 96%), ammonium persulfate (APS) (Amresco), chloroform (Alfa Aesar, 99%), dichloromethane (DCM) (Acros Organics, 99%), ethanol (Decon Laboratories), ethyl ether anhydrous (Fisher Scientific), poly(ethylene glycol) (PEG4K; M_n_ = 4000) (Mallinckrodt), triethylamine (TEA) (Alfa Aesar, 99+%), tetrahydrofuran (THF) (DriSolv), N,N,N',N'-tetramethylethylenediamine (TEMED) (Calbiochem), dimethyl sulfoxide (DMSO) (Fisher Scientific), pentaerythritol tetrakis(3-mercaptopropionate) (PETMP) (Sigma Aldrich, 97%).

DCM and TEA were distilled from CaH_2_ under N_2_ and stored under N_2_ in a dry, air-free flask. All other chemicals were used as received.

### Biological Materials

Fetal bovine serum (Atlanta Biologicals), L-glutamine (Hyclone), MEM Richter's Modification Medium (Hyclone), penicillin streptomycin (MP Biologicals), phosphate buffered saline (PBS) (Corning Cellgro), pure link^TM^ DNase (Invitrogen), pure link^TM^ RNA mini kit (Life Technologies) trypsin/EDTA (Hyclone, 0.05%), TRIzol® (Life Technologies).

### Acrylation of PEG4K

Polyethylene glycol (10 g, 2.5 mmol) (M_n_ 4000) was dissolved in 250 mL THF and triethylamine (1mL, 7.5 mmol) in a round bottom flask and cooled in an ice bath prior to adding acryloyl chloride (470 μL, 5.83 mmol) in 10 mL THF drop-wise to the round bottom flask. The solution was stirred under nitrogen and allowed to warm to room temperature overnight. Reaction was monitored by ^1^H NMR. Triethylamine salts were removed by filtration followed by removal of THF by rotary evaporation. The product poly(ethylene glycol)-4000 diacrylate (PEG4K-DA) was collected by filtration and dried under vacuum overnight. We characterized the product by ^1^H NMR which showed complete acrylation of PEG and no TEA salts. ^1^H NMR spectra (δ ppm) were recorded on a Bruker Biospin Ultrashield 300 MHz NMR Spectrophotometer.

Yield: 58.6%

^1^H NMR (CDCl_3_): δ = 6.43 (d, C**H**_**2**_CH_1_C(O)), δ = 5.71 (d, C**H**_**2**_CH_1_C(O)), δ6.18 (d, CH_2_C**H**_**1**_C(O)), δ = 3.63 (H-4), δ = 4.39 (d, CH_2_C**H**_**1**_C(O)), δ = 3.63 (m, PEG’s-C**H**_**2**_ **-**C**H**_**2**_ **-),** δ = 3.42 (t, PEG-CH_2_C**H**_**2**_OC(O)CH_2_CH_2_).

### Human Mesenchymal Stem Cell Culture

Human mesenchymal stem cells (hMSCs) were obtained from Texas A&M Health Science Centre College of Medicine through a grant obtained from NCRR of the NIH (P40RR017447). Cells were received with no identifying patient information. The cells are obtained from normal, healthy volunteers pre-screened for infectious diseases under a protocol approved by both the Scott & White and Texas A&M University IRBs with written Informed Consent. The frozen cells were recovered overnight and passaged (seeding density: 60 cells/cm^2^) the next day. The cell culture medium used was MEM Richter's Modification medium with 20% Fetal Bovine Serum, 1% L-Glutamine and 1% Penicillin-Streptomycin. Media was changed once every 3 days. Once the cells reached ~80% confluency, they were trypsinized, counted and used for sample preparation (passage 2 hMSCs). For the scope of this project, we were interested in only analyzing the initial effects of free-radicals, UV exposure, and culture systems. Therefore, we did not test the effect of cell age (by using cells in different passage numbers) on free-radical sensitivity, UV exposure, and culture systems. All our sample sets had hMSCs in passage 2.

### 2D cell culture (Day 1)

Freshly trypsinized hMSCs (500,000 cells) were plated in 75 cm^2^ Corning tissue culture flasks with 20 mL fresh media and placed in the incubator.

### Encapsulation of hMSCs in PEG Hydrogels (Day 1)

Optimal cell density for adequate RNA extraction in the hydrogels was determined to be 3,000,000 cells/ 100 μL. Freshly trypsinized cells were centrifuged down to a cell pellet, which was resuspended in fresh medium. Cells were counted using a hemocytometer. Aliquots having 3,000,000 cells were centrifuged in separate tubes at 0.5 rcf for 10 minutes. The resulting cell pellets were encapsulated in hydrogels which were fabricated as described below.

#### Radical Encapsulation

The hMSC cell pellet (3,000,000 cells) was resuspended in PEG4K-DA solution (0.01 g PEG4K-DA in 80 μL PBS) in a microcentrifuge tube. 20 μL of 2.5 M APS and 20 μL of 1.18 M TEMED was added to the cell+PEG4K-DA solution. This resulted in a 10 wt% PEG4K-DA solution with a final concentration of 5 mM APS and 2.5 mM TEMED in PBS. The solution was swiftly mixed and the gels were cast in the caps of 1.5mL microcentrifuge tubes that resulted in cylindrical hydrogels. After 12 minutes the gels were transferred to fresh medium and the medium was changed after 24 hours. Cell viability was quantified using LIVE/DEAD assay. Fluorescence was detected using a Zeiss AxioObserver Inverted Fluorescent microscope equipped with AxioVision software and cells were counted manually.

#### Michael Addition (Non-Radical) Conjugate Addition

Pre-reaction: PETMP was pre-reacted with PEG4K-DA in a 2:1 PETMP to PEG4K-DA molar ratio (0.03 g of PETMP with 0.125 g PEG4K-DA). The pre-reaction was carried out for 2 hours in 1000 μL deuterated dimethyl sulfoxide. Completion of the reaction was monitored via ^1^H NMR. The product was then precipitated into 200 mL cold diethyl ether, filtered, and dried under vacuum. The pre-reaction product (PEG4K-DA-PETMP) was dissolved in 1000 μL PBS. 25 hydrogels were made from this sample, each with a 100 μL volume.

To achieve an equivalent 2:1 PEG4K-DA to PETMP in the final product, 0.375 g of PEG4K-DA was dissolved in 875 μL PBS. To fabricate one hydrogel, 45 μL of the 0.030 M pre-reaction product and 35 μL of 0.10 M PEG4K-DA solution were mixed. The hMSCs pellet (3,000,000 cells) was resuspensed in this solution to fabricate the one hydrogel. 25 μL of 0.8 M TEMED solution was added to obtain a final concentration of 0.2 M TEMED in the polymer solution to catalyze conjugate addition of the samples. This viscous solution was pipetted into the center of a well in a 24-well plate as a single bead. All samples were cured in 24 well plates resulting in cylindrical gels with a flat bottom and domed top. After 7 minutes fresh medium was added to the gels and the medium was changed after 24 hours.

### UV Exposure Conditions (Day 2)

The UV radiation source was a Black Ray UV Bench Lamp 365 nm, 115V 60 Hz, 0.68 Amps (UVP, LLC). Samples were exposed to UV radiation at 365 nm 3.5 mW/cm^2^ intensity for 25 minutes in two increments of 10 minutes and one increment of 5 minutes, with 10 minutes of darkness in between to minimize heat build-up. The intensity was measured and recorded using an ILT950 spectroradiometer (International Light Technologies). The incident light emitted from the lamp was a bell curve from 300nm to 425nm, with the peak at 365nm. The intensity from that block of wavelengths was measured to be 3.5 mW/cm^2^. The total irradiance from UVA wavelengths (321 nm -390 nm) was ~2.4 mW/cm^2^, from visible light (380 nm -780 nm) ~ 8.7 W/cm^2^, from UVB (281 nm—320 nm) 0.025 mW/cm^2^, and UVC (220 nm -280 nm) ~ 0.001 mW/cm^2^. These intensities fluctuate by roughly 10% at any given time, and our intensity of interest is decreased 8% by passing through polystyrene.

### RNA Extraction by TRIzol^®^ Method and Spin Column Purification (Day 3)

Total RNA was extracted using the commercially available TRIzol^®^ guanidinium-phenol based reagent followed by spin column purification using Ambion PureLink kit.

#### Homogenization

2D Samples: Plated cells were trypsinized and centrifuged at 0.5 rcf for 10 minutes. 1000 μL TRIzol^®^ was added to the pellet and was pipette homogenized.

3D Samples: Gels were removed from the medium and washed with PBS. They were coarsely homogenized using a plastic tissue homogenizer and then flash frozen in liquid nitrogen. Each sample was thawed in 1000 μL TRIzol^®^. Fine homogenization was performed on ice using an electric tissue homogenizer (Tissue Tearor, Biospec Products, Inc) for 1 minute at speed 35.

### Phase Separation, Precipitation and Purification

Samples in TRIzol^®^ were incubated at room temperature for 5 minutes before further processing. 200 μL chloroform was added to each sample and incubated at room temperature for 3 minutes before centrifuging at 4°C for 15 minutes 12,000xg for phase separation. The aqueous layer was collected and an equal volume of 70% ethanol was added. This solution was centrifuged multiple times through the spin column with wash buffer I and II (protocol by Life Technologies). DNA impurities were removed with DNase bought commercially. In the final step, RNA was eluted in RNase free ultra pure water and stored at -80°C for further analysis.

### RNA Integrity Analysis prior to gene array

RNA integrity analysis was performed by the UCLA Clinical Microarray Core/JCCC Genomics Shared Resource located at UCLA. Analysis was performed using the Agilent 2100 bioanalyzer. The bioanlayzer generated electropherograms and RNA integrity number (RIN). Samples used for microarray experiments need to have RIN > 7. All our samples had distinct 18s and 28s RNA subunit peaks and average RIN > 9.40.

### Gene array processing and data analysis

Gene array processing and post-processing of data were performed by the UCLA Clinical Microarray Core/JCCC Genomics Shared Resource. We used the Affymetrix human genome U133 Plus 2.0 array, with Affymetrix annotation version na33. The Core used Partek Genomics Suite to identify differentially expressed genes (> 2-fold change, p < 0.05, unless otherwise noted). Gene lists were exported for functional annotation clustering and pathway analysis in two different programs, the Database for Annotation, Visualization, and Integrated Discovery (DAVID v6.7)[23, 24]_and Ingenuity Pathway Analysis (IPA, build version 242990, content version 17199142). When using DAVID for functional annotation clustering, default selections and medium stringency for filtering were used unless otherwise noted, and Benjamini-Hochberg multiple comparison correction p-values were used to determine significance in functional annotation clustering in DAVID. Raw data have been deposited to NCBI’s Gene Expression Omnibus under the series record accession GSE58093.

## Supporting Information

S1 Fig Low differential expression in controls shows repeatability of experiment. The small number of gene changes between months represents variations in cell cycle. Comparisons between months for the same groups have very little change. 25 overlapping genes represent the repeatable difference between months of sample preparation. DAVID functional annotation clustering reveals only 1 significant cluster, Mitosis.
(TIF)

S2 Fig A side view of principle components analysis for all samples. 2D samples (blue = 2D_1_UV, red = 2D_1_, pink = 2D_2_UV, burgundy = 2D_2_), 3D_C_ samples (purple = 3D_C_UV, green = 3D_C_), and 3D_R_ samples (light blue = 3D_R_UV, orange = 3D_R_). The 3D_R_ samples look more dispersed in this view, but not because of the UV samples (light blue). All the 3D_R_ samples could be reflecting the variability imparted by radical polymerization. Arbitrary units on all axes are the same as Fig 2 of the manuscript.
(TIF)

S3 Fig Relative expression levels of CHI3L1 and HSPA6 under all conditions. The two most extreme fold changes due to UV and due to polymerization method were CHI3L1 and HSPA6. Here, their relative changes under all 4 combinations of conditions is diagramed, and shows that CHI3L1 and HSPA6 seem to be anti-correlated. However, they do not share any common canonical pathways.
(TIF)

S1 Note Using a single donor with negligible experimental variation to increase sensitivity. (DOCX)

S2 Note No interaction between UV and radical polymerization. (DOCX)

S3 Note CHI3L1 and HSPA6 reflect changes due to polymerization method, do not reinforce UV exposure. (DOCX)

S1 Table Sample details list. In total, eight sample types were created, with 3 replicates each. The month of preparation corresponding to each group is listed along with the abbreviations used for each.
(DOCX)

S2 Table Inclusion or exclusion of UV samples does not affected enriched pathways due to polymerization method. The top functional annotation clusters from DAVID and KEGG pathways identified by DAVID for comparison of polymerization methods that either include or exclude UV samples. There are no changes to the top cluster for up or downregulated genes, while only significance and enrichment order change for lower clusters for the downregulated genes. Many pathways are not statistically significant.
(DOCX)

S3 Table Top 100 canonical pathways identified by Ingenuity in gene changes between 3D_R_±UV vs. 3D_C_ ±UV. 3030 genes. Results greater than 2-fold change, p < 0.05.
(PDF)

S4 Table Top 100 canonical pathways identified by Ingenuity in gene changes in 3DRUV vs. 3DR. 2006 genes. Results greater than 1.5-fold change, p < 0.05.
(PDF)

S5 Table Genes upregulated during differentiation of hMSC not significant from UV. Genes upregulated during differentiation of hMSC (reviewed in [53]) can be found minimally in some UV comparisons, but not reliably upregulated. Fold changes are reported along with their p-values. In instances where we found multiple listings for the same gene, all the fold changes and p-values are given.
(DOCX)
