# Supplementary figures and images for: Low-Dose, Long-Wave UV Light Does Not Affect Gene Expression of Human Mesenchymal Stem Cells
Source: PLoS One. 2015 Sep 29;10(9):e0139307. doi: 10.1371/journal.pone.0139307 (PMC4587745; doi:10.1371/journal.pone.0139307)

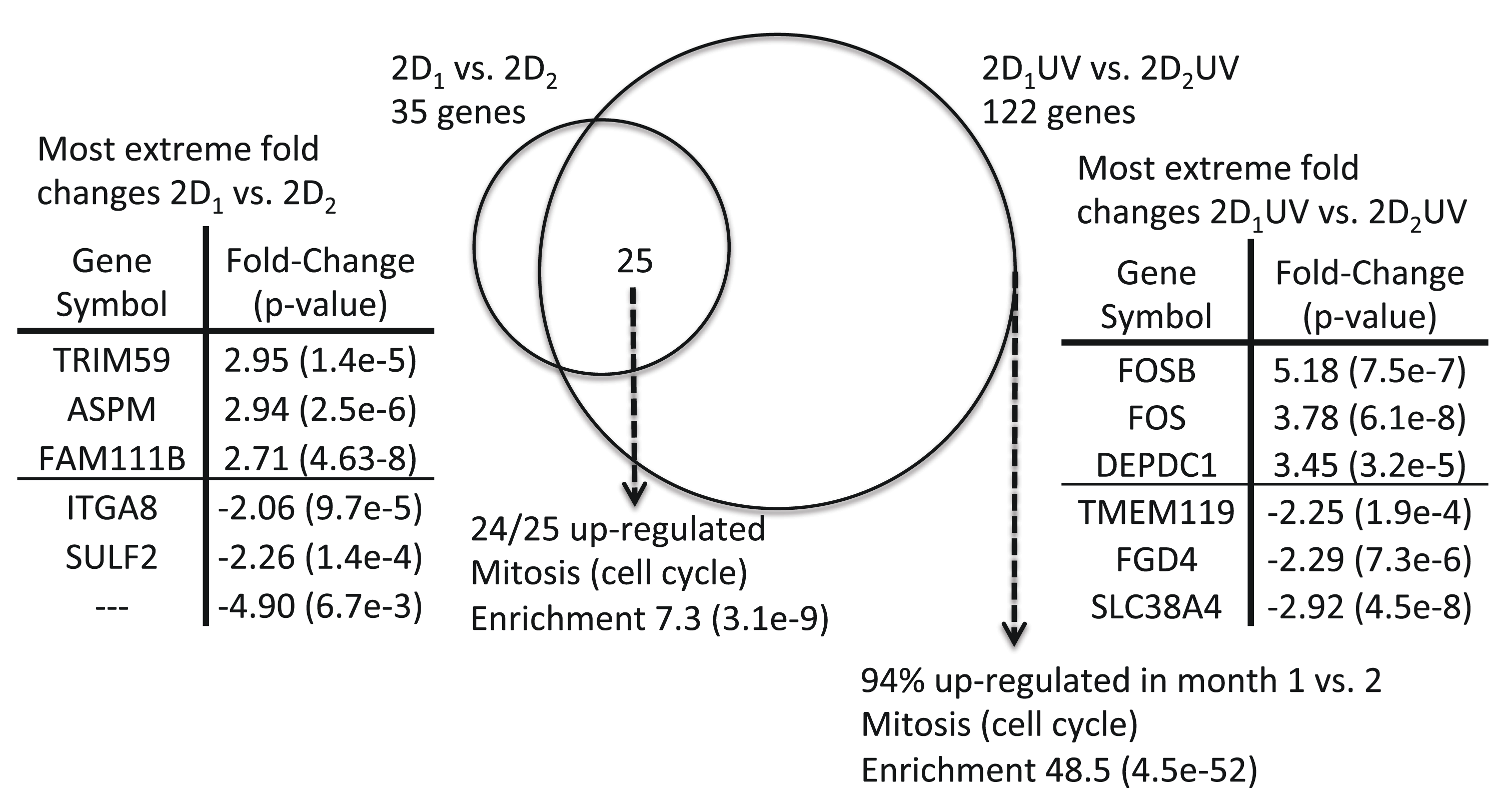

Supplement: S1 Fig — The small number of gene changes between months represents variations in cell cycle. Comparisons between months for the same groups have very little change. 25 overlapping genes represent the repeatable difference between months of sample preparation. DAVID functional annotation clustering reveals only 1 significant cluster, Mitosis. (TIF) [file pone.0139307.s001.tif]

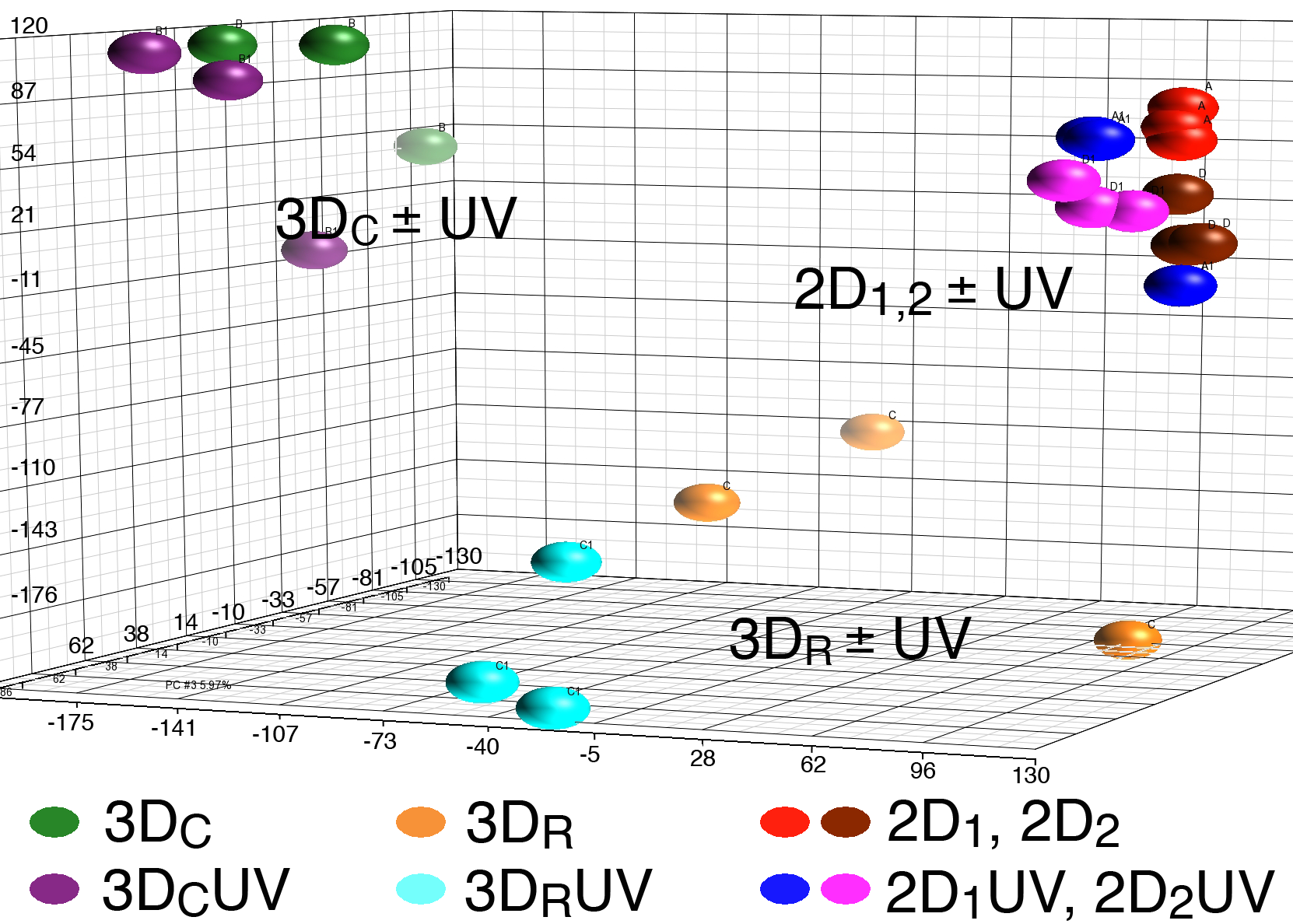

Supplement: S2 Fig — 2D samples (blue = 2D1UV, red = 2D1, pink = 2D2UV, burgundy = 2D2), 3DC samples (purple = 3DCUV, green = 3DC), and 3DR samples (light blue = 3DRUV, orange = 3DR). The 3DR samples look more dispersed in this view, but not because of the UV samples (light blue). All the 3DR samples could be reflecting the variability imparted by radical polymerization. Arbitrary units on all axes are the same as Fig 2 of the manuscript. (TIF) [file pone.0139307.s002.tif]

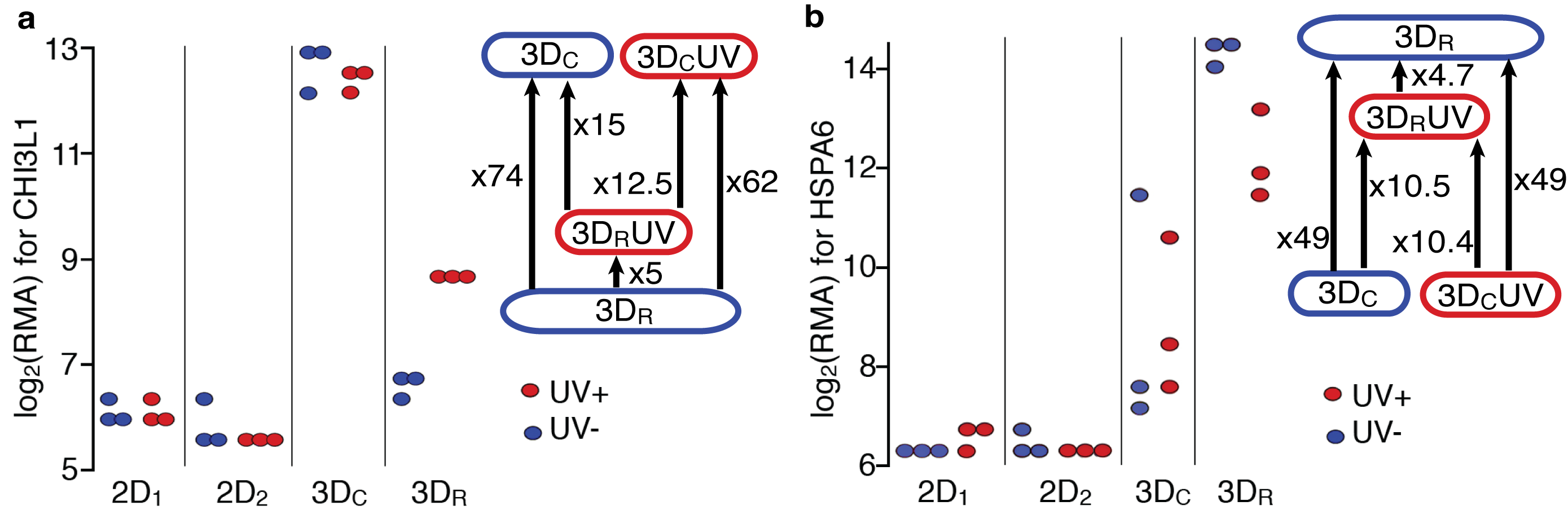

Supplement: S3 Fig — The two most extreme fold changes due to UV and due to polymerization method were CHI3L1 and HSPA6. Here, their relative changes under all 4 combinations of conditions is diagramed, and shows that CHI3L1 and HSPA6 seem to be anti-correlated. However, they do not share any common canonical pathways. (TIF) [file pone.0139307.s003.tif]
