# Supplementary material for: Low-Dose, Long-Wave UV Light Does Not Affect Gene Expression of Human Mesenchymal Stem Cells
Source: PLoS One. 2015 Sep 29;10(9):e0139307. doi: 10.1371/journal.pone.0139307 (PMC4587745; doi:10.1371/journal.pone.0139307)
Supplement: S1 Note — (DOCX) [file pone.0139307.s004.docx]

**Note S1: Using a single donor with negligible experimental variation to increase sensitivity**

2D samples were repeated on two separate dates (n=3 for each date) to establish baseline variations and repeatability of our experimental techniques. We found minimal variations in gene expression between 2D_1_ and 2D_2_, our internal controls, on par with several other studies of hMSC. Briefly, when analyzing 47,000+ transcripts we found low numbers of genes changed (< 0.3%) with low fold changes (< 10 fold) between these control groups, which should be identical aside from the date of expansion. Other research [[1-3](#_ENREF_1)]of hMSC gene expression using the same or similar gene chips to ours reported similarly low variations due to different donors and expansion location (physical laboratory) [[1](#_ENREF_1), [2](#_ENREF_2)], accounting for fewer than 100 differentially expressed genes and most fold changes in the 2-3 fold range but going up to 13 fold. In contrast, hMSC have much greater variability due to changes in cell cycle during routine expansion culture (> 0.9% of genes, up to 59-fold change over 5 days of routine culture)[[3](#_ENREF_3)]. Particularly, differences in gene expression of hMSC between different donors is very minimal[[2](#_ENREF_2)], much less than the differences between sources of hMSC within each donor (bone marrow or adipose) and we use this report to justify using a single donor in our study to amplify any potential changes due to our specific experimental conditions since they may be small. Due to the cost, it is impractical to scan more donors without sacrificing replication in our experimental design. We rely on the base of literature to establish low variability between donors, and based on the many cytotoxicity and control experiments from diverse publications we expect any potential changes due to UV exposure to be small and difficult to detect.

With 3 replicates for each group, we see 35 and 122 unique differentially expressed genes in the 2 comparisons that should have been identical except for the date of their preparation (2D_1_ vs. 2D_2_ and 2D_1_UV vs. 2D_2_UV). In fact, if we look at overlapping genes from those two comparisons, there are merely 25 unique genes and all but one are uniformly upregulated in the first month compared with the second month (Fig S1). CDCA2 was upregulated in the 2D_1_UV vs. 2D_2_UV comparison (2.1 fold) while downregulated in the 2D_1_ vs. 2D_2_ comparison (-2.1 fold). A functional annotation clustering using DAVID reveals only one major annotation cluster that has any significance after multiple comparisons correction. The mitosis (cell cycle) pathway is enriched (enrichment score 7.26, Benjamini 3.1e-9) indicating the two populations were likely in a slightly different part of the cell cycle when harvested for analysis (Fig S1). Thus these small changes do reveal underlying differences and are not necessarily instrumental noise, but rather biological noise or variation. In fact, hMSC from the same passage but at different stages of growth (2 days vs. 7 days after plating) have been shown to differentially upregulate 264 genes and downregulate 208 genes by day 7 (> 2-fold change, p < 0.1).[[3](#_ENREF_3)] That particular study was conducted across 3 different donors using a less stringent p-value cut-off but the same gene array as our study, and observed fold changes as high as 58.6 (*FGF7*), showing that 5 days difference in the same population of cells results in much larger changes to gene expression than we found in any of our 2D comparisons. Comparing overlapping Entrez Gene numbers between their study and our 2D_1_UV vs. 2D_2_UV, we find in particular 22 genes which are all mitosis/cell cycle genes (enrichment 26.5 Benjamini 6.0e-27) which are upregulated in our first month, and also upregulated in their 2 day log phase. If we analyze just the 122 unique differentially expressed genes (only 10 of which were downregulated in month 1 compared to month 2) from 2D_1_UV vs. 2D_2_UV, it becomes apparent that the first month’s samples were collected while more of the cells were undergoing mitosis (enrichment score 48.5, Benjamini 4.5e-52), and that is all the difference between the two months. 2D_1_ vs. 2D_2_ differentially expressed genes were not used for this analysis because there were only 35, too few to create significant enrichment.

References

1. Jansen BJ, Gilissen C, Roelofs H, Schaap-Oziemlak A, Veltman JA, Raymakers RA, et al. Functional differences between mesenchymal stem cell populations are reflected by their transcriptome. Stem Cells Dev. 2010;19(4):481-90. Epub 2009/10/01. doi: 10.1089/scd.2009.0288. PubMed PMID: 19788395.

2. Torensma R, Prins HJ, Schrama E, Verwiel ET, Martens AC, Roelofs H, et al. The impact of cell source, culture methodology, culture location, and individual donors on gene expression profiles of bone marrow-derived and adipose-derived stromal cells. Stem Cells Dev. 2013;22(7):1086-96. Epub 2012/11/14. doi: 10.1089/scd.2012.0384. PubMed PMID: 23145933; PubMed Central PMCID: PMC3608038.

3. Larson BL, Ylostalo J, Prockop DJ. Human multipotent stromal cells undergo sharp transition from division to development in culture. Stem Cells. 2008;26(1):193-201. doi: Doi 10.1634/Stemcells.2007-0524. PubMed PMID: ISI:000252302500022.
