# Supplementary material for: Low-Dose, Long-Wave UV Light Does Not Affect Gene Expression of Human Mesenchymal Stem Cells
Source: PLoS One. 2015 Sep 29;10(9):e0139307. doi: 10.1371/journal.pone.0139307 (PMC4587745; doi:10.1371/journal.pone.0139307)
Supplement: S2 Note — (DOCX) [file pone.0139307.s005.docx]

**Note S2: no interaction between UV and radical polymerization**

Using DAVID for functional annotation clustering and identifying enriched KEGG pathways, the significant clusters remain the same regardless of the inclusion or exclusion of the UV data (S2 Table). The only difference shows up in the downregulated genes, where the clusters are the same, but their relative enrichment scores and p-values are different. Without UV data, Plekstrin homology and WD repeat clusters have significant p-values, but after UV data is added these two clusters become insignificant. Enrichment scores do not change considerably.
