# Supplementary material for: Low-Dose, Long-Wave UV Light Does Not Affect Gene Expression of Human Mesenchymal Stem Cells
Source: PLoS One. 2015 Sep 29;10(9):e0139307. doi: 10.1371/journal.pone.0139307 (PMC4587745; doi:10.1371/journal.pone.0139307)
Supplement: S3 Note — (DOCX) [file pone.0139307.s006.docx]

**Note S3. CHI3L1 and HSPA6 reflect changes due to polymerization method, do not reinforce UV exposure.**

Two genes, *CHI3L1* and *HSPA6*, exhibit the highest fold changes when considering either the effect of UV light (3D_R_ vs. 3D_R_UV and 3D_C_ vs. 3D_C_UV) or the effect of scaffold fabrication (3D_R_±UV vs. 3D_C_ ±UV). *CHI3L1* is downregulated in cells exposed to radicals compared to conjugate addition (3D_C_±UV vs. 3D_R_ ±UV). This implies that radical polymerization does not have the same effect on hMSC *in vitro* as persistent reactive oxygen species in the body, which could cause a cascade of inflammatory events leading to upregulation, translation, and secretion of *CHI3L1* by mesenchymal cells *in vivo*[[1](#_ENREF_1), [2](#_ENREF_2)]. The downregulation of *CHI3L1* in radically polymerized gels may indicate a decrease in stem-ness or a decrease in ECM interaction. Conversely, UV light in the radically polymerized gels seems to induce a very small increase in *CHI3L1* gene expression, but not to a considerable extent (S3 Fig).

*HSPA6*, also known as *Hsp70B’*, is a collagen chaperone that prevents protein aggregation and mediates new protein folding by recognizing and binding net hydrophobic peptide regions that are exposed during translation, membrane translocation, or possibly stress-induced damage. We see this mRNA upregulated with radical polymerization relative to conjugate addition, but down regulated only slightly with UV exposure in 3D_R_. Free radicals are known to induce damage to collagen[[3](#_ENREF_3), [4](#_ENREF_4)], which may explain the upregulation in *HSPA6*. Additionally, if the downregulation of *CHI3L1* due to radical polymerization indicates a decrease in ECM interaction for 3D_R_ compared to 3D_C_, the cell may also be trying to compensate by producing more proteins, whether secreted, intracellular, or transmembrane, and upregulating *HSPA6* in that process. The two genes seem to be anti-correlated with each other, but they have no canonical pathways in common and share only one IPA functional group of little relevance in this context (neurological disease).

References

1. Hoover DJ, Zhu V, Chen R, Briley K, Rameshwar P, Cohen S, et al. Expression of the Chitinase Family Glycoprotein YKL-40 in Undifferentiated, Differentiated and Trans-Differentiated Mesenchymal Stem Cells. PLoS One. 2013;8(5). doi: ARTN e62491

DOI 10.1371/journal.pone.0062491. PubMed PMID: ISI:000319737700014.

2. Prakash M, Bodas M, Prakash D, Nawani N, Khetmalas M, Mandal A, et al. Diverse pathological implications of YKL-40: Answers may lie in 'outside-in' signaling. Cell Signal. 2013;25(7):1567-73. doi: Doi 10.1016/J.Cellsig.2013.03.016. PubMed PMID: ISI:000320638000004.

3. Monboisse JC, Braquet P, Borel JP. Oxygen-Free Radicals as Mediators of Collagen Breakage. Agents Actions. 1984;15(1-2):49-50. doi: Doi 10.1007/Bf01966960. PubMed PMID: WOS:A1984TJ25600024.

4. Monboisse JC, Poulin G, Braquet P, Randoux A, Ferradini C, Borel JP. Effect of Oxy Radicals on Several Types of Collagen. Int J Tissue React. 1984;6(5):385-90. PubMed PMID: WOS:A1984ABB0900005.
