# Supplementary material for: Low-Dose, Long-Wave UV Light Does Not Affect Gene Expression of Human Mesenchymal Stem Cells
Source: PLoS One. 2015 Sep 29;10(9):e0139307. doi: 10.1371/journal.pone.0139307 (PMC4587745; doi:10.1371/journal.pone.0139307)
Supplement: S1 Table — In total, eight sample types were created, with 3 replicates each. The month of preparation corresponding to each group is listed along with the abbreviations used for each. (DOCX) [file pone.0139307.s007.docx]

***Table S1****.* ***Sample details list.*** *In total, eight sample types were created, with 3 replicates each. The month of preparation corresponding to each group is listed along with the abbreviations used for each.*

| Abbreviation | Culture condition | Month of preparation | Parallel UV exposure abbreviation |
| --- | --- | --- | --- |
| 2D_1_ | Polystyrene flask monolayer, | 1^st^ | 2D_1_UV |
| 2D_2_ | Polystyrene flask monolayer, | 2^nd^ | 2D_2_UV |
| 3D_C_ | Conjugate addition encapsulation | 1^st^ | 3D_C_ UV |
| 3D_R_ | Radical polymerization encapsulation | 2^nd^ | 3D_R_UV |
