# Supplementary material for: Low-Dose, Long-Wave UV Light Does Not Affect Gene Expression of Human Mesenchymal Stem Cells
Source: PLoS One. 2015 Sep 29;10(9):e0139307. doi: 10.1371/journal.pone.0139307 (PMC4587745; doi:10.1371/journal.pone.0139307)
Supplement: S2 Table — The top functional annotation clusters from DAVID and KEGG pathways identified by DAVID for comparison of polymerization methods that either include or exclude UV samples. There are no changes to the top cluster for up or downregulated genes, while only significance and enrichment order change for lower clusters for the downregulated genes. Many pathways are not statistically significant. (DOCX) [file pone.0139307.s008.docx]

**Table S2**. **Inclusion or exclusion of UV samples does not affect significant pathways. ^a^**

|  | **DAVID functional annotation clusters** | | **KEGG pathways** | |
| --- | --- | --- | --- | --- |
|  | **Name** | **E=enrichment score (Benjamini-Hochberg)** | **Name** | **[# genes, % of pathway] (Benjamimi-Hochberg)** |
| **Upregulated in**  **3D_R_ ±UV vs 3D_C_±UV**  **(547 unique IDs)** | Mitosis | E: 11.8 (3.86e-12) | Cell Cycle | [18, 3.3%] (1.6e-3) |
|  | Stress response | E: 10.4 (9.19e-13) | p53 signaling pathway | [11, 2.0%] (3.3e-2) |
|  | Intracellular non-membrane-bounded organelle | E: 9.6 (1.38e-8) | MAPK signaling pathway | [23, 4.2%] (8.4e-2) |
|  |  |  | pathways in cancer | [26, 4.6%] (8.5e-2) |
| **Upregulated in**  **3D_R_ vs 3D_C_**  **(830 unique IDs)** | Mitosis | E: 15.8 (7.98e-17) | Cell Cycle | [28, 3.4%] (5.3e-7) |
|  | Stress response | E: 12.1 (2.16e-13) | p53 signaling pathway | [16, 1.9%] (9.2e-4) |
|  | Intracellular non-membrane-bound organelle | E: 10.5 (6.72e-10) | pathways in cancer | [35, 4.2%] (8.1e-2) |
|  |  |  | MAPK signaling pathway | [30, 3.6%] (6.6e-2) |
| **Downregulated in**  **3D_R_±UV vs 3D_C_±UV**  **(1308 unique IDs)** | Cytoskeleton | E: 4.6 (7.07e-4) | Steroid biosynthesis | [8, 0.61%] (9.9e-3) |
|  | Plekstrin homology | E: 3.7 (3.67e-2) | Insulin signaling pathway | [21, 1.61%] (4.2e-2) |
|  | Zinc finger | E: 3.6 (2.66e-4) | Focal adhesion | [26, 2.0%] (7.0e-2) |
|  | WD repeat | E: 3.4 (1.06e-2) |  |  |
| **Downregulated in**  **3D_R_ vs 3D_C_**  **(1624 unique IDs)** | Cytoskeleton | E: 5.4 (4.35e-6) | Steroid biosynthesis | [8, 0.493%] (4.2e-2) |
|  | WD repeat | E: 5.4 (2.15e-4) | Wnt signaling pathway | [25, 1.5%] (9.4e-2) |
|  | Zinc finger | E: 5.3 (3.23e-5) | Focal adhesion | [28, 1.7%] (3.3e-1) |
|  | Plekstrin homology | E: 3.9 (2.34e-5) |  |  |

^a^The top functional annotation clusters from DAVID and KEGG pathways identified by DAVID for comparison of polymerization methods that either include or exclude UV samples. There are no changes to the top cluster for up or downregulated genes, while only significance and enrichment order change for lower clusters for the downregulated genes. Many pathways are not statistically significant.
