# Supplementary material for: Low-Dose, Long-Wave UV Light Does Not Affect Gene Expression of Human Mesenchymal Stem Cells
Source: PLoS One. 2015 Sep 29;10(9):e0139307. doi: 10.1371/journal.pone.0139307 (PMC4587745; doi:10.1371/journal.pone.0139307)
Supplement: S3 Table — Results greater than 2-fold change, p < 0.05. (PDF) [file pone.0139307.s009.pdf]

Ingenuity Canonical Pathways: Top 100 3DR±UV vs. 3DC±UV	-log(p-value	Ratio
Aldosterone Signaling in Epithelial Cells	5.74E00	2.14E-01
IL-6 Signaling	5.12E00	2.34E-01
Mouse Embryonic Stem Cell Pluripotency	5.09E00	2.53E-01
IGF-1 Signaling	4.34E00	2.24E-01
Superpathway of Cholesterol Biosynthesis	4.33E00	1.26E-01
Prolactin Signaling	4.32E00	2.38E-01
Molecular Mechanisms of Cancer	3.97E00	1.5E-01
Integrin Signaling	3.94E00	1.83E-01
Cholesterol Biosynthesis I	3.85E00	1.75E-01
Cholesterol Biosynthesis II (via 24,25-dihydrolanosterol)	3.85E00	1.75E-01
Cholesterol Biosynthesis III (via Desmosterol)	3.85E00	1.75E-01
Role of Macrophages, Fibroblasts and Endothelial Cells in Rheumatoid Arthritis	3.68E00	1.46E-01
Role of Tissue Factor in Cancer	3.56E00	1.85E-01
Insulin Receptor Signaling	3.52E00	1.81E-01
Epithelial Adherens Junction Signaling	3.48E00	1.88E-01
eNOS Signaling	3.37E00	1.68E-01
TR/RXR Activation	3.35E00	1.83E-01
PPARα/RXRα Activation	3.34E00	1.61E-01
JAK/Stat Signaling	3.33E00	2.39E-01
Cholecystokinin/Gastrin-mediated Signaling	3.25E00	2.08E-01
ILK Signaling	3.24E00	1.66E-01
Glucocorticoid Receptor Signaling	3.09E00	1.47E-01
Hepatic Fibrosis / Hepatic Stellate Cell Activation	3.03E00	1.74E-01
Glioblastoma Multiforme Signaling	2.98E00	1.67E-01
Fcγ Receptor-mediated Phagocytosis in Macrophages and Monocytes	2.88E00	1.89E-01
HGF Signaling	2.86E00	1.89E-01
FAK Signaling	2.86E00	1.79E-01
Pancreatic Adenocarcinoma Signaling	2.83E00	1.72E-01
EGF Signaling	2.77E00	2.19E-01
Gap Junction Signaling	2.74E00	1.55E-01
Acute Myeloid Leukemia Signaling	2.74E00	2.02E-01
Acute Phase Response Signaling	2.66E00	1.66E-01
IL-17A Signaling in Airway Cells	2.63E00	1.97E-01
Role of NFAT in Cardiac Hypertrophy	2.59E00	1.48E-01
Regulation of the Epithelial-Mesenchymal Transition Pathway	2.59E00	1.58E-01
PEDF Signaling	2.58E00	2.03E-01
p53 Signaling	2.44E00	1.76E-01
HMGB1 Signaling	2.44E00	1.74E-01
Erythropoietin Signaling	2.43E00	1.9E-01
Actin Cytoskeleton Signaling	2.42E00	1.45E-01
RhoA Signaling	2.42E00	1.8E-01
Glioma Signaling	2.39E00	1.68E-01
Thrombin Signaling	2.38E00	1.52E-01
Growth Hormone Signaling	2.36E00	1.92E-01
Renal Cell Carcinoma Signaling	2.36E00	1.9E-01
Breast Cancer Regulation by Stathmin1	2.34E00	1.5E-01
PI3K Signaling in B Lymphocytes	2.33E00	1.61E-01
ErbB2-ErbB3 Signaling	2.31E00	2.06E-01
VEGF Signaling	2.3E00	1.65E-01
PPAR Signaling	2.24E00	1.68E-01
Role of Osteoblasts, Osteoclasts and Chondrocytes in Rheumatoid Arthritis	2.22E00	1.41E-01
PDGF Signaling	2.22E00	1.86E-01
Remodeling of Epithelial Adherens Junctions	2.21E00	2E-01
Paxillin Signaling	2.19E00	1.62E-01
Small Cell Lung Cancer Signaling	2.18E00	1.6E-01
Reelin Signaling in Neurons	2.16E00	1.88E-01
Semaphorin Signaling in Neurons	2.15E00	2.22E-01
Endometrial Cancer Signaling	2.15E00	2E-01
Endothelin-1 Signaling	2.14E00	1.47E-01
IL-17 Signaling	2.12E00	2E-01
Ovarian Cancer Signaling	2.07E00	1.51E-01
Protein Ubiquitination Pathway	2.07E00	1.44E-01
Clathrin-mediated Endocytosis Signaling	2.06E00	1.52E-01
Leptin Signaling in Obesity	2.06E00	1.76E-01
Role of NANOG in Mammalian Embryonic Stem Cell Pluripotency	2.05E00	1.69E-01
IL-8 Signaling	2.03E00	1.33E-01
ERK/MAPK Signaling	2.03E00	1.42E-01

Melatonin Signaling	2.03E00	1.73E-01
Agrin Interactions at Neuromuscular Junction	2.03E00	2E-01
Gαq Signaling	2.02E00	1.46E-01
Acetate Conversion to Acetyl-CoA	2E00	2.73E-01
CNTF Signaling	1.99E00	1.93E-01
Aryl Hydrocarbon Receptor Signaling	1.99E00	1.35E-01
Colorectal Cancer Metastasis Signaling	1.97E00	1.34E-01
Thrombopoietin Signaling	1.95E00	1.88E-01
Epoxysqualene Biosynthesis	1.95E00	2.22E-01
Taurine Biosynthesis	1.95E00	2.22E-01
Virus Entry via Endocytic Pathways	1.95E00	1.68E-01
DNA Double-Strand Break Repair by Homologous Recombination	1.93E00	2.78E-01
DNA Double-Strand Break Repair by Non-Homologous End Joining	1.93E00	2.5E-01
HER-2 Signaling in Breast Cancer	1.9E00	1.83E-01
TGF-β Signaling	1.9E00	1.78E-01
Cardiac Hypertrophy Signaling	1.9E00	1.37E-01
Type II Diabetes Mellitus Signaling	1.88E00	1.17E-01
Hypoxia Signaling in the Cardiovascular System	1.87E00	1.91E-01
IL-4 Signaling	1.86E00	1.75E-01
IL-3 Signaling	1.81E00	1.87E-01
ErbB Signaling	1.8E00	1.78E-01
NRF2-mediated Oxidative Stress Response	1.77E00	1.44E-01
MSP-RON Signaling Pathway	1.76E00	1.96E-01
Factors Promoting Cardiogenesis in Vertebrates	1.76E00	1.63E-01
AMPK Signaling	1.75E00	1.22E-01
Renin-Angiotensin Signaling	1.74E00	1.51E-01
Ceramide Biosynthesis	1.73E00	1.88E-01
Zymosterol Biosynthesis	1.73E00	1.36E-01
UVA-Induced MAPK Signaling	1.71E00	1.63E-01
SAPK/JNK Signaling	1.71E00	1.52E-01
Prostate Cancer Signaling	1.71E00	1.46E-01
IL-2 Signaling	1.68E00	1.8E-01
B Cell Receptor Signaling	1.67E00	1.43E-01
