# Supplementary material for: Low-Dose, Long-Wave UV Light Does Not Affect Gene Expression of Human Mesenchymal Stem Cells
Source: PLoS One. 2015 Sep 29;10(9):e0139307. doi: 10.1371/journal.pone.0139307 (PMC4587745; doi:10.1371/journal.pone.0139307)
Supplement: S4 Table — Results greater than 1.5-fold change, p < 0.05. (PDF) [file pone.0139307.s010.pdf]

Top 100 Ingenuity Canonical Pathways: 3DrUV vs. 3Dr	-log(p-value	Ratio
Protein Ubiquitination Pathway	8.34E00	1.81E-01
Mitotic Roles of Polo-Like Kinase	4.63E00	2.16E-01
Aldosterone Signaling in Epithelial Cells	4.63E00	1.67E-01
Glucocorticoid Receptor Signaling	3.73E00	1.27E-01
Hepatic Fibrosis / Hepatic Stellate Cell Activation	2.53E00	1.35E-01
NRF2-mediated Oxidative Stress Response	2.43E00	1.28E-01
Cell Cycle: G2/M DNA Damage Checkpoint Regulation	2.18E00	1.84E-01
Sonic Hedgehog Signaling	2.15E00	2E-01
Hereditary Breast Cancer Signaling	2.11E00	1.27E-01
ERK/MAPK Signaling	1.93E00	1.14E-01
Bile Acid Biosynthesis, Neutral Pathway	1.77E00	6.9E-02
Mouse Embryonic Stem Cell Pluripotency	1.77E00	1.41E-01
5-aminoimidazole Ribonucleotide Biosynthesis I	1.73E00	1.11E-01
eNOS Signaling	1.69E00	1.1E-01
Molecular Mechanisms of Cancer	1.68E00	9.82E-02
Pyrimidine Deoxyribonucleotides De Novo Biosynthesis I	1.66E00	1.18E-01
Regulation of the Epithelial-Mesenchymal Transition Pathway	1.62E00	1.12E-01
Aryl Hydrocarbon Receptor Signaling	1.46E00	9.94E-02
Myo-inositol Biosynthesis	1.45E00	2.5E-01
RAN Signaling	1.45E00	1.67E-01
Acute Myeloid Leukemia Signaling	1.42E00	1.31E-01
Estrogen-mediated S-phase Entry	1.4E00	1.79E-01
Cyclins and Cell Cycle Regulation	1.35E00	1.15E-01
NF-κB Signaling	1.3E00	1.09E-01
Basal Cell Carcinoma Signaling	1.3E00	1.3E-01
Role of JAK1 and JAK3 in γc Cytokine Signaling	1.3E00	1.32E-01
IL-9 Signaling	1.28E00	1.5E-01
Methylglyoxal Degradation III	1.27E00	1.3E-01
Small Cell Lung Cancer Signaling	1.23E00	1.06E-01
Pyridoxal 5'-phosphate Salvage Pathway	1.22E00	1.2E-01
DNA damage-induced 14-3-3σ Signaling	1.21E00	1.82E-01
Cleavage and Polyadenylation of Pre-mRNA	1.17E00	2.31E-01
Androgen Biosynthesis	1.17E00	1.15E-01
Prolactin Signaling	1.17E00	1.19E-01
TNFR2 Signaling	1.15E00	1.47E-01
RhoA Signaling	1.12E00	1.15E-01
Superoxide Radicals Degradation	1.1E00	2.5E-01
Glutamate Removal from Folates	1.09E00	3.33E-01
Sulfite Oxidation IV	1.09E00	2.5E-01
PTEN Signaling	1.07E00	1.01E-01
Telomerase Signaling	1.07E00	1.13E-01
Superpathway of Methionine Degradation	1.04E00	7.81E-02
PDGF Signaling	1.04E00	1.16E-01
ILK Signaling	1.04E00	9.76E-02
Netrin Signaling	1.04E00	1.03E-01
ATM Signaling	9.95E-01	1.21E-01
Cell Cycle: G1/S Checkpoint Regulation	9.95E-01	1.18E-01
Role of BRCA1 in DNA Damage Response	9.63E-01	1.13E-01
Activation of IRF by Cytosolic Pattern Recognition Receptors	9.63E-01	1.08E-01
PPAR Signaling	9.6E-01	1.03E-01
Role of JAK2 in Hormone-like Cytokine Signaling	9.51E-01	1.35E-01
Methionine Degradation I (to Homocysteine)	9.37E-01	1.3E-01
PEDF Signaling	9.29E-01	1.14E-01
CD40 Signaling	9.02E-01	1.13E-01
Salvage Pathways of Pyrimidine Ribonucleotides	8.8E-01	9.71E-02
TGF-β Signaling	8.8E-01	1.11E-01

Airway Pathology in Chronic Obstructive Pulmonary Disease	8.79E-01	1.82E-01
Salvage Pathways of Pyrimidine Deoxyribonucleotides	8.79E-01	9.52E-02
Hypoxia Signaling in the Cardiovascular System	8.73E-01	1.18E-01
Oncostatin M Signaling	8.66E-01	1.43E-01
IL-17A Signaling in Gastric Cells	8.58E-01	1.43E-01
Role of JAK family kinases in IL-6-type Cytokine Signaling	8.58E-01	1.43E-01
Role of Osteoblasts, Osteoclasts and Chondrocytes in Rheumatoid Arthritis	8.38E-01	8.84E-02
IL-17A Signaling in Fibroblasts	8.27E-01	1.25E-01
Role of CHK Proteins in Cell Cycle Checkpoint Control	8.14E-01	1.19E-01
γ-linolenate Biosynthesis II (Animals)	8.13E-01	1.25E-01
Mitochondrial L-carnitine Shuttle Pathway	8.13E-01	1.36E-01
Cysteine Biosynthesis III (mammalia)	8.13E-01	1E-01
Antiproliferative Role of TOB in T Cell Signaling	8.12E-01	1.54E-01
GDP-L-fucose Biosynthesis II (from L-fucose)	8.09E-01	1.11E-01
Glycerol-3-phosphate Shuttle	8.09E-01	1.11E-01
RANK Signaling in Osteoclasts	8.08E-01	1.03E-01
Human Embryonic Stem Cell Pluripotency	7.8E-01	8.7E-02
Colorectal Cancer Metastasis Signaling	7.71E-01	8.58E-02
Agrin Interactions at Neuromuscular Junction	7.65E-01	1.14E-01
D-myo-inositol (1,4,5)-trisphosphate Degradation	7.59E-01	1.3E-01
HGF Signaling	7.56E-01	9.91E-02
TNFR1 Signaling	7.53E-01	1.11E-01
IL-10 Signaling	7.4E-01	1.03E-01
Growth Hormone Signaling	7.4E-01	1.03E-01
p38 MAPK Signaling	7.32E-01	1E-01
NAD Phosphorylation and Dephosphorylation	7.19E-01	1.05E-01
GADD45 Signaling	7.1E-01	1.25E-01
Fcγ Receptor-mediated Phagocytosis in Macrophages and Monocytes	6.81E-01	9.43E-02
Chronic Myeloid Leukemia Signaling	6.81E-01	9.43E-02
p53 Signaling	6.62E-01	9.26E-02
Role of PKR in Interferon Induction and Antiviral Response	6.59E-01	1.02E-01
GM-CSF Signaling	6.56E-01	1.03E-01
Ubiquinol-10 Biosynthesis (Eukaryotic)	6.54E-01	6.9E-02
Purine Nucleotides De Novo Biosynthesis II	6.54E-01	4.76E-02
1,25-dihydroxyvitamin D3 Biosynthesis	6.51E-01	9.09E-02
Ascorbate Recycling (Cytosolic)	6.51E-01	7.69E-02
Glutathione Redox Reactions II	6.51E-01	1.43E-01
S-adenosyl-L-methionine Biosynthesis	6.51E-01	1.25E-01
IL-6 Signaling	6.43E-01	9.68E-02
CD27 Signaling in Lymphocytes	6.42E-01	1.02E-01
Role of RIG1-like Receptors in Antiviral Innate Immunity	6.3E-01	1.02E-01
Pancreatic Adenocarcinoma Signaling	6.25E-01	8.59E-02
Ovarian Cancer Signaling	6.14E-01	8.55E-02
Bladder Cancer Signaling	6.06E-01	9.28E-02
