# Supplementary material for: Low-Dose, Long-Wave UV Light Does Not Affect Gene Expression of Human Mesenchymal Stem Cells
Source: PLoS One. 2015 Sep 29;10(9):e0139307. doi: 10.1371/journal.pone.0139307 (PMC4587745; doi:10.1371/journal.pone.0139307)
Supplement: S5 Table — Genes upregulated during differentiation of hMSC (reviewed in [53]) can be found minimally in some UV comparisons, but not reliably upregulated. Fold changes are reported along with their p-values. In instances where we found multiple listings for the same gene, all the fold changes and p-values are given. (DOCX) [file pone.0139307.s011.docx]

***Table S5****.* ***Genes upregulated during differentiation of hMSC*** *(reviewed in [*[*1*](#_ENREF_1)*]) can be found minimally in some UV comparisons, but not reliably upregulated. Fold changes are reported along with their p-values. In instances where we found multiple listings for the same gene, all the fold changes and p-values are given.*

| **Lineage** | **Gene Symbol** | **2D_1+2_UV vs. 2D_1+2_**  **Fold chg** | **3D_C_UV vs. 3D_C_**  **Fold chg**  **(p-value)** | **3D_R_UV vs. 3D_R_**  **Fold chg**  **(p-value)** | **3D_R_±UV vs. 3D_C_±UV**  **Fold chg (p-value)** |
| --- | --- | --- | --- | --- | --- |
| Osteo | *CRYAB* | --- | --- | --- | 2.44 (1.32e-8) |
|  | *ID4* | --- | --- | -2.00 (5.50e-3)  -2.03 (3.11e-3) | 3.95 (5.30e-9)  2.83 (4.17e-6)  2.04 (1.27e-8) |
| Chond | *ADAMTS5* | --- | --- | 2.04 (1.44e-2) | -4.87 (2.11e-7)  -3.95 (5.77e-8)  -3.82 (9.46e-7) |
| Adipo | *PDK4*  *ZEB* | --- | --- | --- | 7.78( 5.22e-7)  2.06 (3.22e-5) |
|  |  | --- | --- | --- | [ZEB1]-2.54 (2.38e-9)  -3.71(7.29e-8)  [ZEB2]-2.08 (1.30e-8) |
| Neuro | *NEGR1* | --- | -2.76 (1.43e-5)  -2.63 (5.57e-5)  -2.52 (1.69e-4) | -2.55 (7.67e-5)  -2.47 (5.29e-5) | --- |
|  | *SOCS2* | --- | --- | --- | 2.14 (1.33e-5) |

References

1. Menicanin D, Bartold PM, Zannettino ACW, Gronthos S (2009) Genomic Profiling of Mesenchymal Stem Cells. Stem Cell Reviews and Reports 5: 36-50.
